# Alcohol exposure promotes cell communication network 1 cleavage in esophageal adenocarcinoma cells exclusively

**DOI:** 10.1002/ccs3.70103

**Published:** 2026-08-05

**Authors:** Wula Aladan, Baoxin Chi, Zhiheng Chang, Mizhu Wang, Tong Dang, Xianmei Meng, Jianyuan Chai

**Affiliations:** ^1^ Inner Mongolia Institute of Digestive Diseases The Second Affiliated Hospital of Baotou Medical College Inner Mongolia University of Science and Technology Baotou China; ^2^ The First Hospital of Huhhot Huhhot China

**Keywords:** alcohol, CCN1, esophageal cancer, MMP2, MMP9

## Abstract

Alcohol consumption has been a risk factor for more than 200 diseases, including esophageal cancer. Cell communication network 1 (CCN1), a matricellular protein, is highly expressed in esophageal squamous cell carcinoma (ESCC) but is barely detectable in esophageal adenocarcinoma (EAC). Alcohol consumption has been identified as a major contributor to ESCC development, but its role in EAC is uncertain. This study examines the impact of acute (30 min) or prolonged (12 h) alcohol exposure (5, 100, and 500 mM) on CCN1 expression and function in esophageal epithelial cells, including normal (HEEC), ESCC (KYSE150 and KYSE410), and EAC (OE19 and OE33), in association with the activity of MMP2, MMP9, and MMP14. It was found that alcohol exposure promoted CCN1 expression in both normal and tumor cells but induced CCN1 cleavage exclusively in EAC cells, generating an 18‐kDa fragment that promotes tumor growth. MMP9, which was active only in EAC cells, was found to mediate this cleavage. Forced activation of MMP9 in either normal or ESCC cells improved cell viability, whereas inhibition of MMP9 in EAC cells attenuated cell survival. Taken together, alcohol exposure promotes MMP9‐mediated CCN1 cleavage in EAC, converting CCN1 from a pro‐death to a pro‐survival factor for EAC. This makes alcohol consumption a risk factor not only for ESCC development but also for EAC progression. A preprint can be found at d197for5662m48.cloudfront.net.

## Graphical abstract

An illustration shows how cell communication network 1 (CCN1) is involved in the normal esophagus, esophageal squamous cell carcinoma (ESCC), and esophageal adenocarcinoma (EAC) after alcohol exposure. Alcohol exposure upregulates CCN1 expression in the esophageal epithelium, with or without esophageal cancer. In normal individuals, CCN1 repairs tissue damage inflicted by alcohol and accelerates the healing process. However, if alcohol exposure continues repeatedly, esophageal inflammation becomes chronic, which may lead to ESCC. In this case, CCN1 overexpression may facilitate tumor growth by promoting cell proliferation and migration. In the case of patients with EAC, alcohol consumption not only upregulates CCN1 expression but also promotes CCN1 cleavage via MMP9 activation. Overexpression of CCN1 may promote tumor cell death, but CCN1 cleavage benefits tumor cell survival. Therefore, alcohol consumption is harmful to all individuals, including patients with esophageal cancer.

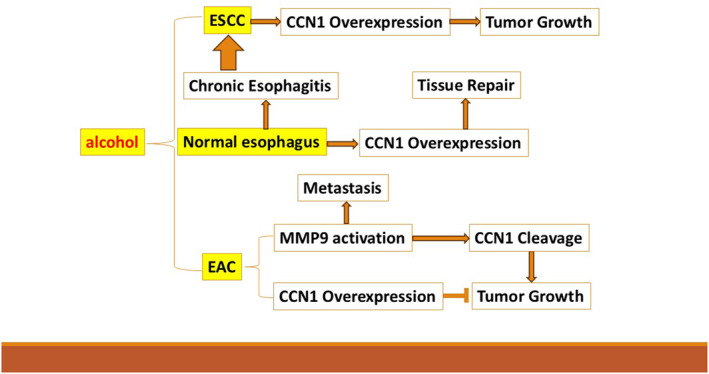

## 1 BACKGROUND

Alcohol is one of the most commonly abused substances worldwide, especially in Eastern Europe and Eastern Asia.^1^ Although Eastern Europe has made remarkable progress in cutting down alcohol consumption per capita in recent years, the situation in Eastern Asia has gotten worse, particularly in China, India, and Vietnam, where the alcohol market has expanded by 104% from 1990 to 2017.^2^ Alcohol consumption is causally related to >200 diseases, including several types of cancer, for example, head/neck cancer, esophageal cancer, liver cancer, colorectal cancer, breast cancer, and others.^3^, ^4^, ^5^ As shown in the year 2020, nearly 4.1% of new cancer cases were attributable to alcohol consumption, among which esophageal cancer was at the top, responsible for 25.59% of all cases.^6^

The majority of cases of esophageal cancer are diagnosed as either esophageal squamous cell carcinoma (ESCC) or esophageal adenocarcinoma (EAC), in an approximately 9:1 ratio. ESCC development has been directly linked to alcohol consumption, particularly when the drinker is also a tobacco smoker.^7^, ^8^, ^9^ In China, offering alcoholic beverages and cigarettes is customarily viewed as two inseparable friendly gestures. “It is not a banquet without alcohol served,” as the locals say. Therefore, alcohol and cigarettes are commonly provided at most social gatherings as a custom. This observation might explain why >50% of the global cases of esophageal cancer are found in this country. Despite the predominance of ESCC, EAC diagnoses have been rising consistently over the past few decades,^10^, ^11^ becoming a growing concern globally.

Cell communication network 1 (CCN1/Cyr61) is a matricellular protein that primarily supports cell adhesion, proliferation, and migration, playing a critical role in multiple physiological and pathological processes, such as angiogenesis, wound healing, inflammation, and tumorigenesis.^12^, ^13^, ^14^, ^15^ Although some cancers (e.g., breast cancer and pancreatic cancer) take advantage of CCN1 to facilitate tumor growth and metastasis, others (e.g., endometrial cancer and non‐small‐cell lung carcinoma) are suppressed by CCN1. Esophageal cancer is an excellent example. CCN1 is highly expressed in ESCC but barely detectable in EAC. Consequently, CCN1 has been found to promote ESCC tumor growth but induces EAC cell death by apoptosis.^16^, ^17^

Metalloproteinases (MMPs) are known to facilitate cancer cell invasion and metastasis by digesting the extracellular matrix (ECM).^18^ Alcohol exposure has been documented to increase MMP2 and MMP9 expression and activity in various types of cells, such as hepatocytes, where it also promotes CCN1 expression.^19^, ^20^, ^21^, ^22^ As a component of the ECM, CCN1 contains multiple MMP digestion sites. It can be cleaved by MMP2 or MMP14 into three fragments: 29, 18, and 8 kDa, among which the 18‐kDa form has been found to stimulate cell proliferation more potently than the full‐length CCN1 (42 kDa) in retinal endothelial cells.^23^

This study compared CCN1 expression and function in esophageal cancer cells to those of normal epithelial cells in the context of alcohol exposure.

## 2 MATERIALS AND METHODS

### 2.1 Cell culture, transfection, and reagents

Human EAC cell lines OE19 (RRID: CVCL_1622) and OE33 (RRID: CVCL_0471) (MilliporeSigma) and ESCC cell lines KYSE150 (RRID: CVCL_1348) and KYSE410 (RRID: CVCL_1352) (BeNa Culture Collection) were cultured in the Roswell Park Momerial Institute medium (Thermo Fisher Scientific) supplemented with 10% Fetal bovine serum (FBS) (MilliporeSigma). The human esophageal epithelial cell line HEEC (RRID: CVCL_3702) (BeNa Culture Collection) was maintained in Dulbecco's Modified Eagle Medium (Thermo Fisher Scientific) supplemented with 10% FBS. An equivalent number of cells was plated in 6‐well plates and cultured to 80% confluency for subsequent experiments. The cell cycle was then synchronized in the basal medium for 6 h before treatments. Cells were treated with 5‐, 100‐, or 500‐mM alcohol for either 30 min or 12 h. To inhibit MMP2 or MMP9 activity, cells were preincubated with 100 μM MMP2 inhibitor I (MilliporeSigma, #444244) or 100 μM MMP9 inhibitor II (MilliporeSigma, #444293), respectively, for 1 h before treatment with ethanol. To activate MMP9, 4‐aminophenylmercuric acetate (APMA) (MilliporeSigma, #A9563) was used at 1 mM. To knock down MMP9 or CCN1, cells were transfected with a pRS vector carrying an shRNA specifically against MMP9 (OriGene Technologies, #TR311436) or CCN1 (OriGene, TR313575) using Lipofectamine LTX PLUS (Thermo Fisher Scientific), following the manufacturer's protocol. The control cells were transfected with the same vector carrying a negative sequence (5′‐TACCAGAGCTAACTCAGATAGTACT‐3′). Assays were done 48 h after transfection. All the cell lines used in this study were authenticated periodically and have not been previously reported as misidentified or contaminated, including mycoplasma contamination. The cells were replaced every five passages with new stocks supplied by the manufacturers to ensure their authenticity.

### 2.2 Cell‐free CCN1 cleavage detection assay

Recombinant MMP9 (rMMP9) (R&D Systems, #911MP) was diluted to 100 μg/mL in assay buffer (50 mM Tris, 10 mM CaCl_2_, 150 mM NaCl, 0.05% Brij 35, pH 7.5). To activate MMP9, APMA was added to a final concentration of 1 mM. The mixture was incubated at 37°C for 24 h and then further diluted to 0.4 μg/mL in the assay buffer. Recombinant full‐length CCN1 (OriGene, #TP750169) was diluted to 0.5 μg/mL in the assay buffer. The active MMP9 solution was combined with the CCN1 solution in a 1:1 ratio and incubated at 37°C for 2 h. The reaction products were boiled in the sample loading buffer for 5 min, separated by Sodium Dodecyl Sulfate Polyacrylamide Gel Electrophoresis (SDS‐PAGE), and stained with Coomassie blue R‐250 solution (MilliporeSigma).

### 2.3 Protein isolation and Western blotting

After treatment, total protein was extracted from cells using a modified radioimmunoprecipitation assay lysis buffer containing 50 mM Tris‐HCl (pH 7.5), 150 mM NaCl, 5 mM EDTA, 0.5% sodium deoxycholate, and 1% NP‐40. The supernatant was collected after centrifugation at 4°C, 10,000 × g for 10 min. Conventional Western blot analysis was performed. Briefly, equivalent amounts of protein samples (30–50 μg each) were resolved by SDS‐PAGE at 100 V and transferred to a nitrocellulose membrane at 390 mA for 90 min. Subsequently, the membrane was blocked with 5% nonfat milk for 1 h at room temperature and then incubated with a primary antibody for 2 h at room temperature on a shaker. After three 10‐min washes with Tris Buffered Saline with Tween‐20 buffer, the membrane was incubated with an appropriate HRP‐conjugated secondary antibody (Santa Cruz Biotechnology) on a shaker for 1 h. The signal was developed in enhanced chemillumunescence solution (Amersham Pharmacia), and images were captured and quantified using Gel Doc XR (Bio‐Rad Laboratories). β‐Actin was used as the loading control. The data were collected from multiple independent experiments and analyzed for statistical significance. The following primary antibodies were used: CCN1 (OriGene, #TA349858), Cyr61 (Santa Cruz Biotechnology, #sc‐271217), Cyr61 (LSBio, #LS‐C392953), MMP2 (OriGene, #TA378583), MMP9 (OriGene, #TA378586), MMP14 (OriGene, #TA332551), and β‐actin (Santa Cruz Biotechnology, #sc69879). Recombinant human MMP2 (Boster, #PROT08253 and #PROT08253‐4), MMP9 (Novus, #911‐MP and 11602‐MP), and MMP14 (OriGene, #TP308917) were used in Western blotting to determine the sizes of the targets.

### 2.4 Zymography

Gelatinase activity was assessed using cell lysates and conditioned cell culture media. Briefly, protein samples in nonreducing sample buffer (0.4 M Tris, pH 6.8, 5% SDS, 20% glycerol, 0.03% bromophenol blue) were separated on 10% SDS‐PAGE gels containing 1 mg/mL gelatin (Thermo Fisher Scientific). After electrophoresis, gels were washed in 2.5% Triton X‐100 with gentle shaking for 30 min and then incubated for 30 min at room temperature in the developing buffer (40 mM Tris‐HCl, 0.2 mM NaCl, 6.67 mM CaCl_2_, 0.1% Triton X‐100, pH 7.8). Next, the gel was incubated overnight in fresh developing buffer at 37°C. Next, the gel was stained for 2 h in a 0.5% Coomassie blue R‐250 solution containing 10% acetic acid and 40% methanol and then destained by washing with a 45% ethanol and 10% acetic acid solution three times (15 min per wash). The gel was kept in a 5% methanol and 0.75% acetic acid solution for storage and imaging. The active MMPs appeared as white bands at the appropriate molecular weight marker on a dark blue background.

### 2.5 Cell viability assay

Cell Counting Kit 8 (CCK‐8; MilliporeSigma, #96992) assays were used to assess cell viability after the indicated treatments according to the manufacturer’s protocol. Briefly, an equivalent number of cells was distributed per well in a 96‐well plate at 100 μL/well. The plate was incubated at 37°C and 5% CO_2_ for 24 h and then switched to the serum‐free medium for 6 h. After the indicated treatments, 10 μL of CCK‐8 reagent was added to each well of the cell culture. The plate was then placed back in the incubator for a further 4 h. Cell viability was measured using a plate reader at 450 nm.

### 2.6 Animal study

The animal experiments were approved by the Institutional Animal Care and Use Committee of the Second Affiliated Hospital of Baotou Medical College (BMC2021‐81960445DS) and performed following the ARRIVE Guidelines for the use of animals in experiments. A total of 10 male Sprague–Dawley rats with body weights ranging from 225 to 250 g were purchased from Saibainuo Biotech Company. The animals were allowed to acclimatize to the conditions at the facility (20–22°C, 60%–80% relative humidity, 12‐h light/dark cycle) for 7 days before the experiment commenced. Animals were fasted overnight, and the following day, 5 rats were randomly selected to drink 40% ethanol instead of water every other day. The ethanol concentration used was based on being comparable to that of regular liquor. The remaining 5 rats were given regular water as a control. The animal health was monitored daily by the facility staff. No animals were found dead during the experiment. All rats were euthanized 4 weeks later by intraperitoneal injection of pentobarbital at 200 mg/kg body weight, followed by exsanguination. The esophagus was removed for histological study. This method is consistent with the ARRIVE recommendations.

### 2.7 Immunohistochemistry

Tissue specimens were fixed in 10% formalin at room temperature overnight and embedded in paraffin. Tissue sections were deparaffinized and rehydrated, and antigen retrieval was performed in a microwave oven by pause heating. Slides were stained with hematoxylin and eosin for regular histological analysis or incubated with the primary antibody of interest for 2 h in a humidified chamber. After 3 × 10 min washes in phosphate‐buffered saline, signals were developed using an LSAB+ kit (Agilent Technologies) containing a universal secondary linker and HRP‐conjugated streptavidin with aminoethyl carbazole chromogen as the substrate. Nuclei were counterstained with hematoxylin.

### 2.8 Statistical analysis

All numerical data are presented as the mean ± standard deviation and were analyzed using single‐classification one‐way ANOVA. *p* < 0.05 was considered to indicate a statistically significant difference.

## 3 RESULTS

### 3.1 Alcohol consumption induces esophagitis and CCN1 overexpression

To determine how alcohol consumption affected esophageal CCN1 expression in vivo, drinking water for the experimental group of rats was replaced with 40% ethanol every other day for 4 weeks. This ethanol concentration was equivalent to that of common liquors on the market. The control group was given regular water. As shown in Figure 1, alcohol consumption resulted in severe inflammation in the esophagus, characterized by epithelial thickening and elongation of the lamina propria into the epithelium. Along with the development of esophagitis, epithelial CCN1 expression was highly upregulated (Figure S1).

**FIGURE 1:**
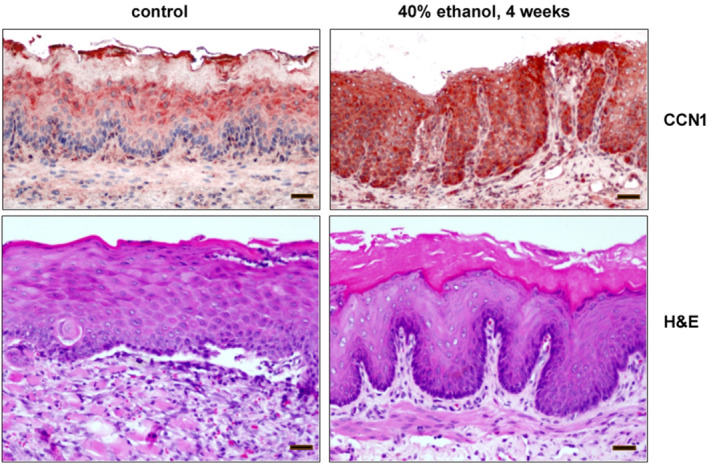
The esophageal tissue of rats after 4 weeks of drinking 40% alcohol or water (control). Upper panel—immunohistochemistry with a CCN1 antibody (OriGene, #TA349858) shows increased CCN1 expression in the esophageal epithelium after alcohol consumption; lower panel—H&E staining shows increased esophageal epithelial thickness after alcohol consumption, with an inflammatory reaction. Scale bar, 10 μm. CCN1, cell communication network 1; H&E, hematoxylin and eosin.

### 3.2 Alcohol exposure upregulates CCN1 expression in both normal and cancerous esophageal cells but increases CCN1 cleavage only in EAC cells

To determine the mechanisms behind the CCN1 upregulation in alcohol‐induced esophagitis, we performed in vitro experiments using esophageal epithelial cells, including normal (HEEC), ESCC (KYSE150 and KYSE410), and EAC (OE19 and OE33). The following three concentrations of ethanol were used in our experiments: 5 mM, 100 mM, and 500 mM. The experimental conditions were selected based on the consideration of alcohol intoxication in humans. The definition of driving under the influence of alcohol varies from country to country. A driver can be arrested in the United States when their blood alcohol content (BAC) is >80 mg/100 mL (or 17.36 mM), whereas in China, this limit is 20 mg/100 mL (or 4.34 mM), and in India, it is 40 mg/100 mL (or 8.68 mM). However, it is considered potentially fatal when the BAC is >400 mg/100 mL (or 86.8 mM) regardless of geographical region. Some extreme cases are documented in the literature, among which individuals have survived with a BAC >1500 mg/100 mL (∼250 mM).

After a 6‐h synchronization in serum‐free media, cells were incubated in the ethanol‐containing medium for 30 min or 12 h. Total protein was extracted from the cells to examine CCN1 expression. As shown in Figure 2 and Tables 1, 2, 3, full‐length CCN1 (42 kDa) was upregulated in all three cell types, whereas an 18‐kDa isoform of CCN1 was found only in EAC cells and substantially increased in response to alcohol exposure.

**FIGURE 2:**
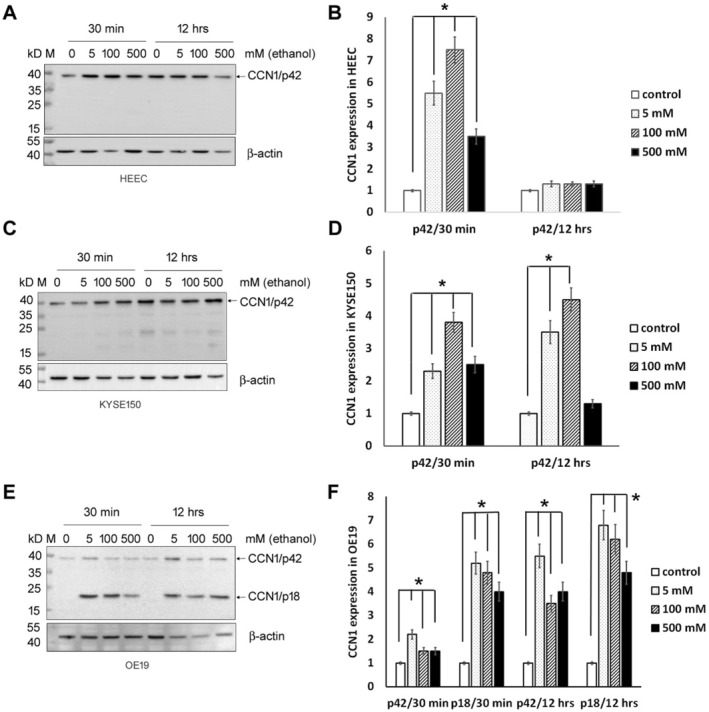
CCN1 expression in HEEC, KYSE150, and OE19 cells after incubation with different concentrations of ethanol (5, 100, and 500 mM) for 30 min or 12 h. (A) Western blot analyses of CCN1 expression in HEEC cells normalized to β‐actin. (B) Quantitative analysis of CCN1 expression in alcohol‐treated HEEC cells compared to the control. (C) Western blot analyses of CCN1 expression in KYSE150 cells normalized to β‐actin. (D) Quantitative analysis of CCN1 expression in alcohol‐treated KYSE150 cells compared to the control. (E) Western blot analyses of CCN1 expression in OE19 cells normalized to β‐actin. (F) Quantitative analysis of CCN1 expression in alcohol‐treated OE19 cells compared to the control. The antibody for CCN1 was from OriGene (#TA349858). The quantitative analyses were based on five replicates. *Statistical significance at *p* < 0.01. CCN1, cell communication network 1.

**TABLE 1:** Fold changes of CCN1 expression in response to alcohol exposure in HEEC cells, based on five Western blotting experiments.

HEEC	5 mM	100 mM	500 mM
CCN1/p42, 30 min	5.5 ± 1.2	7.5 ± 1.6	3.5 ± 0.8
CCN1/p42, 12 h	1.3 ± 0.2	1.3 ± 0.2	1.3 ± 0.2
CCN1/p18, 30 min	—	—	—
CCN1/p18, 12 h	—	—	—

Abbreviation: CCN1, cell communication network 1.

**TABLE 2:** Fold changes of CCN1 expression in response to alcohol exposure in KYSE150 cells, based on five Western blotting experiments.

KYSE150	5 mM	100 mM	500 mM
CCN1/p42, 30 min	2.3 ± 0.5	3.8 ± 1.1	2.5 ± 0.8
CCN1/p42, 12 h	3.5 ± 0.7	4.5 ± 1.2	1.3 ± 0.2
CCN1/p18, 30 min	—	—	—
CCN1/p18, 12 h	—	—	—

Abbreviation: CCN1, cell communication network 1.

**TABLE 3:** Fold changes of CCN1 expression in response to alcohol exposure in OE19 cells, based on five Western blotting experiments.

OE19	5 mM	100 mM	500 mM
CCN1/p42, 30 min	3.1 ± 1.0	1.5 ± 0.5	1.8 ± 0.5
CCN1/p42, 12 h	5.2 ± 1.2	4.8 ± 1.2	4.0 ± 1.0
CCN1/p18, 30 min	5.5 ± 1.2	3.5 ± 1.3	4.0 ± 0.2
CCN1/p18, 12 h	6.8 ± 1.5	6.2 ± 1.5	4.8 ± 1.3

Abbreviation: CCN1, cell communication network 1.

### 3.3 Alcohol exposure promotes MMP9 activation in EAC cells only

CCN1 has been previously reported to be a substrate of MMP2 and MMP14, and both cleave CCN1, producing an 18‐kDa peptide.^23^ We wondered whether the 18‐kDa fragment seen in EAC cells was the same one reported in the literature and how alcohol exposure affected the expression of these two MMPs. Because MMP9 shares many characteristics with MMP2, we included MMP9 in the study as well. Based on the amino acid composition, the molecular weights of MMP2, MMP14, and MMP9 are predicted to be 74 kDa (MMP2/pro), 66 kDa (MMP14/pro), and 78 kDa (MMP9/pro), respectively. However, due to post‐translational modifications, they often appear larger in SDS‐PAGE. These enzymes become active by removing an ∼10‐kDa fragment from the N terminus. As shown in Figure 3, MMP2 was found to be constitutively active in all three types of cells, particularly in ESCC, where active MMP2 was the only detectable form, regardless of alcohol exposure (Figure 3C,D). MMP9, on the other hand, was activated only in EAC cells, especially in the presence of alcohol (Figure 3E,F). It coincided with the increase in CCN1 expression and cleavage observed above (Figure 2E,F), making us wonder whether CCN1 in EAC cells might have been cleaved by MMP9, rather than MMP2, because MMP14 expression was undetectable in any cell lines used in this study. Zymography confirmed the exclusive activation of MMP9 in EAC cells after alcohol exposure (Figure 4A). Similar results were seen in vivo, where MMP9 was inactive in the esophageal epithelium regardless of alcohol uptake (Figure S1).

**FIGURE 3:**
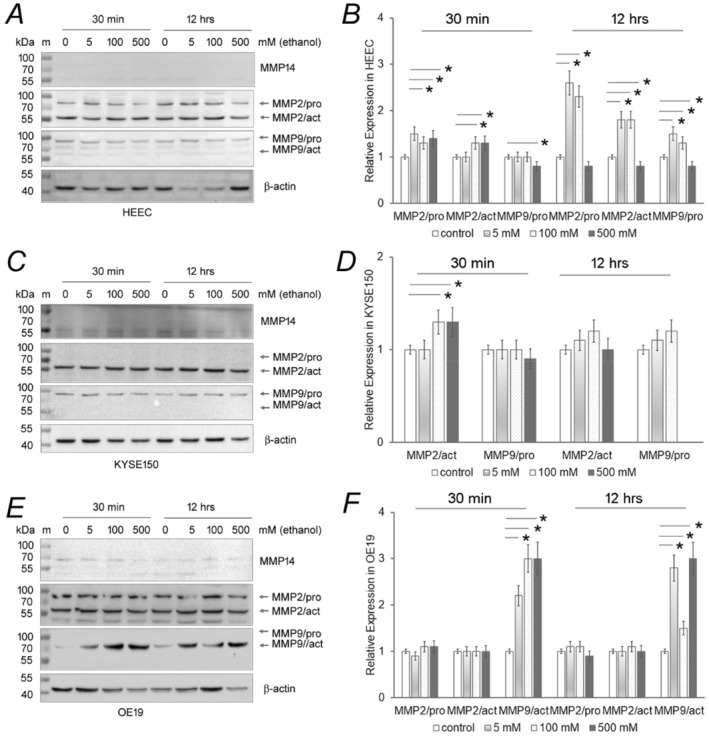
MMP2, MMP9, and MMP14 expression in HEEC, KYSE150, and OE19 cells after incubation with different concentrations of ethanol (5, 100, and 500 mM) for 30 min or 12 h. (A) Western blot analyses of MMP2, MMP9, and MMP14 expression in HEEC cells normalized to β‐actin. (B) Quantitative analysis of MMP2 and MMP9 expression in alcohol‐treated HEEC cells compared to the control. MMP14 expression was unquantifiable. (C) Western blot analyses of MMP2, MMP9, and MMP14 expression in KYSE150 cells normalized to β‐actin. (D) Quantitative analysis of MMP2 and MMP9 expression in alcohol‐treated KYSE150 cells compared to the control. MMP14 expression was unquantifiable. (E) Western blot analyses of MMP2, MMP9, and MMP14 expression in OE19 cells normalized to β‐actin. (F) Quantitative analysis of MMP2 and MMP9 expression in alcohol‐treated OE19 cells compared to the control. MMP14 expression was unquantifiable. All the quantitative analyses were based on at least 5 replicates. The antibodies for MMP2, MMP9, and MMP14 were from OriGene, with catalog numbers #TA378583, #TA378586, and #TA332551, respectively. *Statistical significance at *p* < 0.01.

**FIGURE 4:**
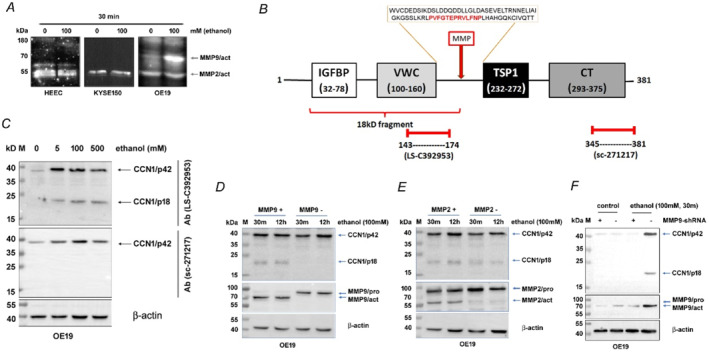
CCN1 cleavage in EAC cells. (A) Zymography of MMPs. After alcohol stimulation, the conditioned cell culture media were collected and separated by 10% SDS‐PAGE containing 1 mg/mL gelatin and stained with a 0.5% Coomassie blue R‐250 solution. The active MMPs appear as white bands at the appropriate molecular weight marker on a dark background. (B) Scheme of the CCN1 protein structure with a notion of possible MMP cleavage site. (C) After 30‐min exposure of OE19 cells to various concentrations of ethanol, protein was extracted from the cells for Western blot analysis. Two CCN1 antibodies were used: The LS‐C392953 antibody recognizes the VWC region of CCN1, and the sc‐271217 antibody detects the CT region. (D) OE19 cells were preincubated with MMP9 inhibitor II before alcohol exposure. Protein was isolated from the cells for Western blot analyses of CCN1 (#TA349858) and MMP9 (#TA378586), normalized to β‐actin. (E) OE19 cells were preincubated with MMP2 inhibitor I before alcohol exposure. Protein was isolated from the cells for Western blot analyses of CCN1 and MMP2, normalized to β‐actin. (F) MMP9 in OE19 cells was knocked down with shRNA transfection. The expression of CCN1 (#TA349858) and MMP9 (#TA378586) in response to alcohol exposure was assessed by Western blot analyses. CCN1, cell communication network 1; CT, C‐terminal cysteine‐knot region; EAC, esophageal adenocarcinoma; MMPs, metalloproteinases; SDS‐PAGE, Sodium Dodecyl Sulfate Polyacrylamide Gel Electrophoresis; VWC, von Willebrand factor Type C.

### 3.4 MMP9 mediates alcohol‐augmented CCN1 cleavage

The full‐length CCN1 consists of 381 amino acids across four domains: insulin‐like growth factor binding protein (IGFBP), von Willebrand factor Type C (VWC), thrombospondin Type 1 (TSP1), and C‐terminal cysteine‐knot region (CT). Based on a previous study,^23^ MMP2 and MMP14 can both cleave CCN1 between the VWC and TSP1 domains, generating an 18‐kDa functional peptide containing the IGFBP and VWC domains (Figure 4B). To determine whether the CCN1 fragment seen in EAC cells was the one reported previously, we used two antibodies to detect CCN1: LS‐C392953 targeting amino acids 143–174 (VWC region) and sc‐271217 targeting amino acids 345–381 (CT region). As shown in Figure 4C, after cells were exposed to alcohol for 30 min, LS‐C392953 detected both forms of CCN1 (42 and 18 kDa), whereas sc‐271217 was only able to detect the full‐length CCN1 protein, indicating that the CCN1 cleavage site in EAC cells was very likely the same one recognized by either MMP2 or MMP14.^23^

To clarify whether MMP2 or MMP9 was responsible for CCN1 cleavage in EAC cells, we incubated the cells with a specific MMP9 inhibitor (MMP9 inhibitor II) before alcohol exposure. As shown in Figure 4D, the 18‐kDa form of CCN1 completely disappeared in the presence of the inhibitor. However, when the same experiment was repeated using an MMP2 inhibitor, the 18‐kDa fragment remained (Figure 4E). The role of MMP9 in CCN1 cleavage was also demonstrated by knocking down MMP9 in OE19 cells (Figure 4F). To verify this result, we used a cell‐free system in which a full‐length recombinant CCN1 protein was incubated with rMMP9 at 37°C for 2 h in the presence or absence of APMA, a potent MMP9 activator. As shown in Figure 5A, in the presence of APMA, the CCN1 protein was cleaved into two bands, 18 and 24 kDa. Taken together, these results provide evidence that CCN1 in EAC cells was cleaved by MMP9 rather than MMP2, but likely at a site common to both, between the IGFBP and VWC domains.

**FIGURE 5:**
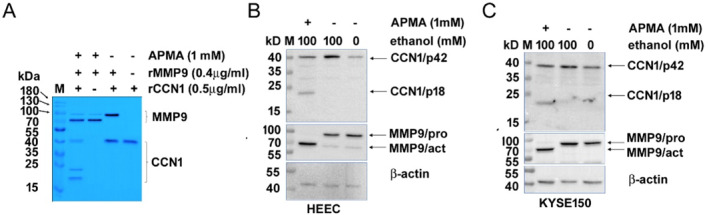
MMP9 is responsible for CCN1 cleavage in esophageal cells. (A) Cell‐free assay of MMP9 and CCN1 interaction. A full‐length rCCN1 protein (#TP750169) was incubated at 1 μg/mL with rMMP9 (#911MP) at 0.4 μg/mL at 37°C for 2 h in the presence or absence of APMA. The gel was stained with 0.5% Coomassie blue R‐250 solution. (B) HEEC cells were preincubated with APMA before alcohol exposure. Protein was isolated from the cells for Western blot analyses of CCN1 (#TA349858) and MMP9 (#TA378586), normalized to β‐actin. (C) KYSE150 cells were preincubated with APMA before alcohol exposure. Protein was isolated from the cells for Western blot analyses of CCN1 (#TA349858) and MMP9 (#TA378586), normalized to β‐actin. APMA, 4‐aminophenylmercuric acetate; CCN1, cell communication network 1; rCCN1, recombinant CCN1; rMMP9, recombinant MMP9.

Because MMP9 was found to be inactive in both normal (Figure 3A,B) and ESCC cells (Figure 3C,D), and CCN1 was not cleaved in these cells either (Figure 2A–D), we decided to test our hypothesis again using these cells. To do so, we incubated KYSE150 and HEEC cells with APMA before alcohol exposure. As shown in Figure 5B (HEEC) and Figure 5C (KYSE150), activation of MMP9 in either normal or ESCC cells resulted in CCN1 cleavage, confirming that activation of MMP9, rather than MMP2, was responsible for the generation of the 18‐kDa form of CCN1 in esophageal cells.

### 3.5 CCN1 cleavage benefits esophageal cell survival

The 18‐kDa fragment of CCN1 was previously reported to be a more potent stimulator for endothelial cell proliferation.^23^ We wondered whether CCN1 cleavage in EAC cells also had this property. For this purpose, we treated cells with 100 mM alcohol for 30 min or 12 h in the presence or absence of the MMP9 inhibitor in 96‐well plates and assessed cell proliferation by CCK‐8 assays. As shown in Figure 6A, OE19 cell viability was increased in response to alcohol exposure but was attenuated by MMP9 inhibition (MMP9−), suggesting that MMP9 activity was critical for EAC cell survival in the presence of alcohol. To confirm the role of CCN1 cleavage in the improved EAC cell viability upon alcohol exposure, OE19 cells were transfected with shRNA against CCN1 and then exposed to alcohol. As shown in Figure 6B, CCN1 deficiency attenuated cell viability, suggesting that CCN1 availability was essential for EAC cell survival in the presence of alcohol. Because MMP9 was inactivated in both normal (Figure 3A,B) and ESCC cells (Figure 3C,D), we added APMA, the MMP9 activator, to the culture medium of KYSE150 and HEEC cells before alcohol treatment. As shown in Figure 6C,D, activation of MMP9 significantly improved the viability of both normal and ESCC cells (*p* < 0.05), indicating that the 18‐kDa CCN1 fragment facilitates esophageal cell growth.

**FIGURE 6:**
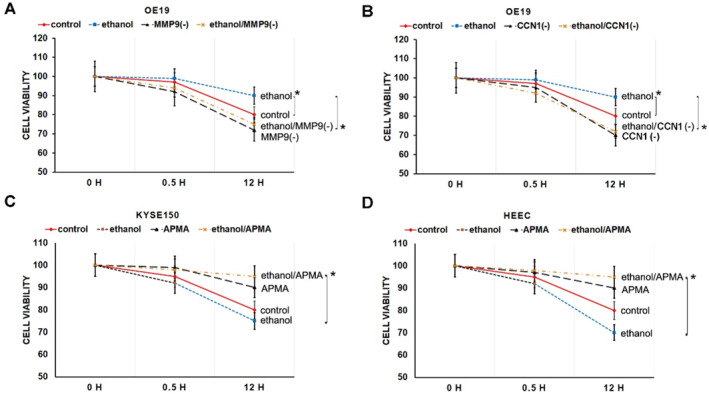
CCN1 cleavage benefits EAC cell growth. (A) OE19 cell viability was assayed after incubation with ethanol at 100 mM for 30 min or 12 h in the presence or absence of MMP9 inhibitor II (MMP9−). (B) CCN1 was suppressed in OE19 cells by shRNA transfection, and cell viability was assayed after incubation with ethanol at 100 mM for 30 min or 12 h. (C) KYSE150 cell viability was assayed after incubation with ethanol at 100 mM for 30 min or 12 h in the presence or absence of APMA. (D) HEEC cell viability was assayed using CCK‐8 after incubation with ethanol at 100 mM for 30 min or 12 h in the presence or absence of APMA. APMA, 4‐aminophenylmercuric acetate; CCK‐8, Cell Counting Kit 8; CCN1, cell communication network 1; EAC, esophageal adenocarcinoma.

## 4 DISCUSSION

CCN1 has been one of our primary research targets for years, with a particular focus on its role in the digestive system^16^. To date, we have found that CCN1 expression is generally low in the epithelial linings of the gastrointestinal tract under physiological circumstances but shoots up tremendously during injury (e.g., ulcers) or after exposure to hazardous insults (e.g., acid and alcohol) and stays high until the condition improves, after which it returns to low expression levels. During this process, CCN1 functions as a mediator, defending epithelial integrity and restoring tissue homeostasis by regulating cell proliferation/migration and interacting with other matrix molecules. EAC development is such a good example. The primary cause of EAC is gastroesophageal reflux disease (GERD), which initially induces esophagitis and then leads to esophageal metaplasia (Barrett’s esophagus) if the refluxate contains bile. Barrett’s esophagus is a precancerous condition of EAC. During the process of tumorigenesis, epithelial CCN1 in the esophagus rises to very high levels at the beginning (esophagitis) and declines gradually if the GERD episodes continue but still remains higher in the metaplastic esophageal epithelium than in normal tissue. Once EAC occurs, however, CCN1 essentially vanishes from the malignant esophageal mucosa.^16^, ^17^ In other words, CCN1 must go down for EAC to grow. Compared to EAC, ESCC does not downregulate CCN1 expression; instead, it upregulates CCN1 to support its tumor growth and metastasis.

Metastasis is a significant challenge of any cancer, and it requires the participation of MMPs to dissolve the ECM, allowing cancer cells to escape from the initial tumor and invade other organs. There are 23 MMPs in humans, which can process a variety of bioactive molecules, including cytokines, chemokines, growth factors, receptors, and ECM components. The functions of these MMPs, however, are highly redundant. For instance, MMP1, MMP8, and MMP13 all cleave collagens at one specific site. The two gelatinases, MMP2 and MMP9, also share several characteristics, both preferring to cleave between glycine and hydrophobic residues within a hydroxyproline bracket. In most organs, MMP2 is constitutively expressed, whereas MMP9 is inducible, and both degrade gelatin and remodel the ECM during wound healing, inflammation, and cancer invasion. They are abundantly expressed in the gastric epithelium,^24^, ^25^ liver fibrosis,^20^ and lung tissue^26^ after alcohol consumption.

Based on PROSPER analysis, the full‐length CCN1 protein contains at least 10 potential cleavage sites for MMPs and thus can be cleaved by multiple MMPs theoretically.^23^ MMP2^27^ and MMP14^28^ have been reported to cleave CCN1 at the same site between the VWC and TSP1 domains, both producing an 18‐kDa fragment that promotes cell proliferation and migration. This cleavage product was also seen in our study, exclusively in EAC cells, but was not associated with either MMP2 or MMP14 activity. Instead, it was produced by MMP9 activation. Based on analysis using antibodies targeting the VWC and CT regions individually, this cleavage likely occurs between amino acids 207 and 219 (PVFGTEPRVLFNP), the hinge region between the VWC and TSP1 domains. In agreement with the previous study,^23^ our study also showed that expression of this 18‐kDa fragment of CCN1 benefited cell growth, suggesting that alcohol exposure can convert CCN1 from an anti‐EAC agent into a pro‐EAC agent. Based on this notion, drinking alcohol not only increases the risk of ESCC development but also likely advances EAC progression.

Alcohol consumption is a leading risk factor for global diseases and death. According to the Global Burden of Diseases, Injuries, and Risk Factors Study, there were 100.4 million cases of alcohol‐related diseases and 3 million alcohol‐related deaths in 2016,^1^, ^29^ and the incidence continues to grow year after year. According to a recent investigation,^30^ >50% of males in China can be classified into the hazardous or harmful drinking categories, and this trend is predicted to grow by another 17% in the next few years.^2^ This is reflected not only by the growing drinking population but also, more significantly, by the quantity of alcohol consumed, which grows 3 times faster than the rise in the drinking population. No doubt, increased alcohol uptake increases the overall risk of death. According to a theoretical analysis, if alcohol drinking can be completely stopped by 2030, the incidence of cancer in China alone would drop by 11.49% for males and 1.00% for females, among which esophageal cancer incidence would decrease by 25.78%.^30^ That would be a theoretical reduction of 5,382,668 cases of cancer.

We are fully aware of the limitations of this study. First of all, it is primarily based on cell culture, and therefore, the findings may not reflect the true esophageal condition after alcohol consumption. There are no animal models currently available for alcohol‐induced esophageal cancer. The purpose of the animal component in this study was to learn how alcohol consumption changes esophageal expression of CCN1 in vivo, thereby establishing the basis for the rest of the in vitro work. To fill the gap from bench to bed, studies using clinical specimens are inevitable. Meanwhile, a viable animal model can also be useful for elucidating the molecular connections between alcohol consumption and esophageal cancer. Secondly, some mysteries that arose from this study remain troubling. For example, why is MMP9 active only in EAC cells, especially after alcohol exposure, but not in ESCC or normal esophageal epithelial cells? A digestive cut in CCN1 by MMP9 should supposedly produce two fragments; why is the 18‐kDa fragment the only product detectable in cells, whereas two fragments (18‐kDa and 24‐kDa) are visualized in a cell‐free setting? Investigation continues.

## 5 CONCLUSIONS

This study finds that alcohol exposure promotes MMP9‐mediated cleavage of CCN1 in EAC cells exclusively, thereby benefiting tumor cell survival. According to our previous studies,^16^, ^17^ overexpression of CCN1 in EAC cells leads to apoptosis. In contrast, this study shows that alcohol exposure can change the nature of CCN1, converting it from a pro‐death to a pro‐survival factor for EAC. This makes alcohol consumption a risk factor not only for ESCC development but also for EAC progression.

## AUTHOR CONTRIBUTIONS

Wula Aladan, Baoxin Chi, and Zhiheng Chang conducted most of the experimental work, prepared the data, and drafted the manuscript. Mizhu Wang participated in some experiments. Xianmei Meng and Tong Dang provided the resources, funding, and administrative support. Jianyuan Chai conceptualized the study, curated the results, and finalized the manuscript.

## CONFLICT OF INTEREST STATEMENT

The authors declare no conflicts of interest.

## ETHICS STATEMENT

The animal component of the study was approved by the Institutional Animal Care and Use Committee at the Second Affiliated Hospital (BMC2021‐81960445DS). This study adhered to the Declaration of Helsinki.

## CONSENT TO PUBLISH DECLARATION

Not applicable.

## Supporting information

Supporting Information S1

Figure S1: Original images of Western blots.

## Data Availability

Data will be available upon reasonable request from Dr. Chai.

## References

[1] GBD 2016 Alcohol Collaborators . 2018. “Alcohol Use and Burden for 195 Countries and Territories, 1990–2016: A Systematic Analysis for the Global Burden of Disease Study 2016.” Lancet 392(10152): 1015–1035. 10.1016/S0140-6736(18)31310-2. 30146330 PMC6148333

[2] Manthey, J. , K. D. Shield , M. Rylett , O. S. M. Hasan , C. Probst , and J. Rehm . 2019. “Global Alcohol Exposure Between 1990 and 2017 and Forecasts Until 2030: A Modeling Study.” Lancet 393(10190): 2493–2502. 10.1016/S0140-6736(18)32744-2. 31076174

[3] Baan, R. , K. Straif , Y. Grosse , B. Secretan , F. El Ghissassi , V. Bouvard , A. Altieri , V. Cogliano , and WHO International Agency for Research on Cancer Monograph Working Group . 2007. “Carcinogenicity of Alcoholic Beverages.” Lancet Oncology 8(4): 292–293. 10.1016/s1470-2045(07)70099-2. 17431955

[4] Connor, J. 2017. “Alcohol Consumption as a Cause of Cancer.” Addiction 112(2): 222–228. 10.1111/add.13477. 27442501

[5] Rehm, J. , G. Gmel Sr , O. S. M. Hasan , S. Imtiaz , S. Popova , C. Probst , M. Roerecke , et al. 2017. “The Contribution of Unrecorded Alcohol to Health Harm.” Addiction 112(9): 1687–1688. 10.1111/add.13865. 28635161

[6] Rumgay, H. , K. Shield , H. Charvat , P. Ferrari , B. Sornpaisarn , I. Obot , F. Islami , V. E. P. P. Lemmens , J. Rehm , and I. Soerjomataram . 2021. “Global Burden of Cancer in 2020 Attributable to Alcohol Consumption: A Population‐Based Study.” Lancet Oncology 22(8): 1071–1080. 10.1016/S1470-2045(21)00279-5. 34270924 PMC8324483

[7] Liu, Y. , H. Chen , Z. Sun , and X. Chen . 2015. “Molecular Mechanisms of Ethanol‐Associated Oro‐Esophageal Squamous Cell Carcinoma.” Cancer Letters 361(2): 164–173. 10.1016/j.canlet.2015.03.006. 25766659 PMC4765374

[8] Katada, C. , T. Yokoyama , T. Yano , K. Kaneko , I. Oda , Y. Shimizu , H. Doyama , et al. 2016. “Alcohol Consumption and Multiple Dysplastic Lesions Increase Risk of Squamous Cell Carcinoma in the Esophagus, Head, and Neck.” Gastroenterology 151(5): 860–869.e7. 10.1053/j.gastro.2016.07.040. 27492616

[9] Chang, J. , W. Tan , Z. Ling , R. Xi , M. Shao , M. Chen , Y. Luo , et al. 2017. “Genomic Analysis of Oesophageal Squamous‐Cell Carcinoma Identifies Alcohol Drinking‐Related Mutation Signature and Genomic Alterations.” Nature Communications 8(1): 15290. 10.1038/ncomms15290. PMC5477513 28548104

[10] Quante, M. , T. A. Graham , and M. Jansen . 2018. “Insights into the Pathophysiology of Esophageal Adenocarcinoma.” Gastroenterology 154(2): 406–420. 10.1053/j.gastro.2017.09.046. 29037468

[11] Smyth, E. C. , J. Lagergren , R. C. Fitzgerald , F. Lordick , M. A. Shah , P. Lagergren , and D. Cunningham . 2017. “Oesophageal Cancer.” Nature Reviews Disease Primers 3(1): 17048. 10.1038/nrdp.2017.48. PMC6168059 28748917

[12] Yeger, H. , and B. Perbal . 2021. “The CCN Axis in Cancer Development and Progression.” Journal of Cell Communication and Signaling 15(4): 491–517. 10.1007/s12079-021-00618-2. 33877533 PMC8642525

[13] Lau, L. F. 2012. “CCN1 and CCN2: Blood Brothers in Angiogenic Action.” Journal of Cell Communication and Signaling 6(3): 121–123. 10.1007/s12079-012-0169-z. 22833463 PMC3421018

[14] Chen, C. C. , K. H. Kim , and L. F. Lau . 2016. “The Matricellular Protein CCN1 Suppresses Hepatocarcinogenesis by Inhibiting Compensatory Proliferation.” Oncogene 35(10): 1314–1323. 10.1038/onc.2015.190. 26028023 PMC4666840

[15] Ki‐Hyun Kim, K. H. , J. H. Won , N. Cheng , and L. F. Lau . 2018. “The Matricellular Protein CCN1 in Tissue Injury Repair.” Journal of Cell Communication and Signaling 12(1): 273–279. 10.1007/s12079-018-0450-x. 29357009 PMC5842204

[16] Chang, Z. , T. Dang , X. Meng , and J. Chai . 2022. “The Role of CCN1 in Esophageal Adenocarcinoma: What We Have Learned from the Lab.” Cancer Control 29: 10732748221074734. 10.1177/10732748221074734. 35291889 PMC8935545

[17] Dang, T. , C. Modak , X. Meng , J. Wu , R. Narvaez , and J. Chai . 2017. “CCN1 Sensitizes Esophageal Cancer Cells to TRAIL‐Mediated Apoptosis.” Experimental Cell Research 361(1): 163–169. 10.1016/j.yexcr.2017.10.015. 29055676

[18] Kessenbrock, K. , V. Plaks , and Z. Werb . 2010. “Matrix Metalloproteinases: Regulators of the Tumor Microenvironment.” Cell 141(1): 52–67. 10.1016/j.cell.2010.03.015. 20371345 PMC2862057

[19] Phillips, P. A. , J. A. McCarroll , S. Park , M. J. Wu , R. Pirola , M. Korsten , J. S. Wilson , and M. V. Apte . 2003. “Rat Pancreatic Stellate Cells Secrete Matrix Metalloproteinases: Implications for Extracellular Matrix Turnover.” Gut 52(2): 275–282. 10.1136/gut.52.2.275. 12524413 PMC1774949

[20] Kornek, M. , E. Raskopf , I. Guetgemann , M. Ocker , S. Gerceker , M. A. Gonzalez‐Carmona , C. Rabe , T. Sauerbruch , and V. Schmitz . 2006. “Combination of Systemic Thioacetamide (TAA) Injections and Ethanol Feeding Accelerates Hepatic Fibrosis in C_3_H/He Mice and is Associated with Intrahepatic up Regulation of MMP‐2, VEGF and ICAM‐1.” Journal of Hepatology 45(3): 370–376. 10.1016/j.jhep.2006.03.017. 16780996

[21] Chung, A. W. , H. H. Yang , M. K. Sigrist , G. Brin , E. Chum , W. A. Gourlay , and A. Levin . 2009. “Matrix Metalloproteinase‐2 and ‐9 Exacerbate Arterial Stiffening and Angiogenesis in Diabetes and Chronic Kidney Disease.” Cardiovascular Research 84(3): 494–504. 10.1093/cvr/cvp242. 19617223

[22] Jiang, R. , J. Tang , X. Zhang , Y. He , Z. Yu , S. Chen , J. Xia , J. Lin , and Q. Ou . 2022. “CCN1 Promotes Inflammation by Inducing IL‐6 Production via alpha6beta1/PI3K/Akt/NF‐kappaB Pathway in Autoimmune Hepatitis.” Frontiers in Immunology 13: 810671. 10.3389/fimmu.2022.810671. 35547732 PMC9084230

[23] Choi, J. , A. Lin , E. Shrier , L. F. Lau , M. B. Grant , and B. Chaqour . 2013. “Degradome Products of the Matricellular Protein CCN1 as Modulators of Pathological Angiogenesis in the Retina.” Journal of Biological Chemistry 288(32): 23075–23089. 10.1074/jbc.M113.475418. 23798676 PMC3743481

[24] Chakraborty, S. , S. Stalin , N. Das , S. T. Choudhury , S. Ghosh , and S. Swarnakar . 2012. “The Use of Nano‐Quercetin to Arrest Mitochondrial Damage and MMP‐9 Upregulation During Prevention of Gastric Inflammation Induced by Ethanol in Rat.” Biomaterials 33(10): 2991–3001. 10.1016/j.biomaterials.2011.12.037. 22257724

[25] Swarnakar, S. , A. Mishra , K. Ganguly , and A. V. Sharma . 2007. “Matrix Metalloproteinase‐9 Activity and Expression is Reduced by Melatonin During Prevention of Ethanol‐Induced Gastric Ulcer in Mice.” Journal of Pineal Research 43(1): 56–64. 10.1111/j.1600-079X.2007.00443.x. 17614836

[26] Lois, M. , L. A. Brown , I. M. Moss , J. Roman , and D. M. Guidot . 1999. “Ethanol Ingestion Increases Activation of Matrix Metalloproteinases in Rat Lungs During Acute Endotoxemia.” American Journal of Respiratory and Critical Care Medicine 160(4): 1354–1360. 10.1164/ajrccm.160.4.9811060. 10508828

[27] Dean, R. A. , and C. M. Overall . 2007. “Proteomics Discovery of Metalloproteinase Substrates in the Cellular Context by iTRAQ Labeling Reveals a Diverse MMP‐2 Substrate Degradome.” Molecular & Cellular Proteomics 6(4): 611–623. 10.1074/mcp.M600341-MCP200. 17200105

[28] Butler, G. S. , R. A. Dean , E. M. Tam , and C. M. Overall . 2008. “Pharmacoproteomics of a Metalloproteinase Hydroxamate Inhibitor in Breast Cancer Cells: Dynamics of Membrane Type 1 Matrix Metalloproteinase‐Mediated Membrane Protein Shedding.” Molecular and Cellular Biology 28(15): 4896–4914. 10.1128/MCB.01775-07. 18505826 PMC2493375

[29] Millwood, I. Y. , P. K. Im , D. Bennett , P. Hariri , L. Yang , H. Du , C. Kartsonaki , et al. 2023. “Alcohol Intake and Cause‐Specific Mortality: Conventional and Genetic Evidence in a Prospective Cohort Study of 512 000 Adults in China.” The Lancet Public Health 8(12): e956–e967. 10.1016/S2468-2667(23)00217-7. 38000378 PMC7615754

[30] Song, S. , L. Lei , H. Liu , F. Yang , N. Li , W. Chen , J. Peng , and J. Ren . 2023. “Impact of Changing the Prevalence of Smoking, Alcohol Consumption and Overweight/Obesity on Cancer Incidence in China from 2021 to 2050: A Simulation Modelling Study.” EClinicalMedicine 63: 102163. 10.1016/j.eclinm.2023.102163. 37662518 PMC10468350

