# Supplementary material for: Alcohol exposure promotes cell communication network 1 cleavage in esophageal adenocarcinoma cells exclusively
Source: J Cell Commun Signal. 2026 Aug 5;20(3):e70103. doi: 10.1002/ccs3.70103 (PMC13439744; doi:10.1002/ccs3.70103)
Supplement: Supplementary file 1 [file CCS3-20-e70103-s002.pdf]

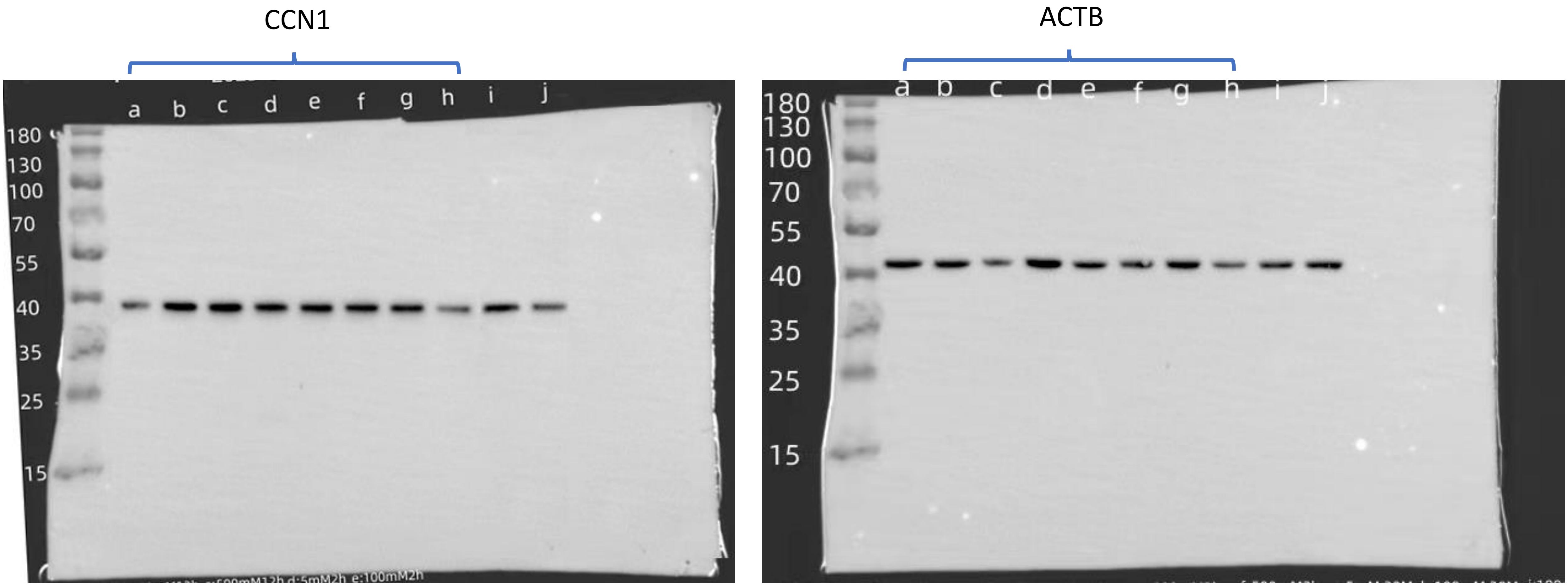

Originals in Fig. 2A. Western blot analyses of CCN1 expression in HEEC cells normalized to β-actin. The antibody for CCN1 was from OriGene (#TA349858).

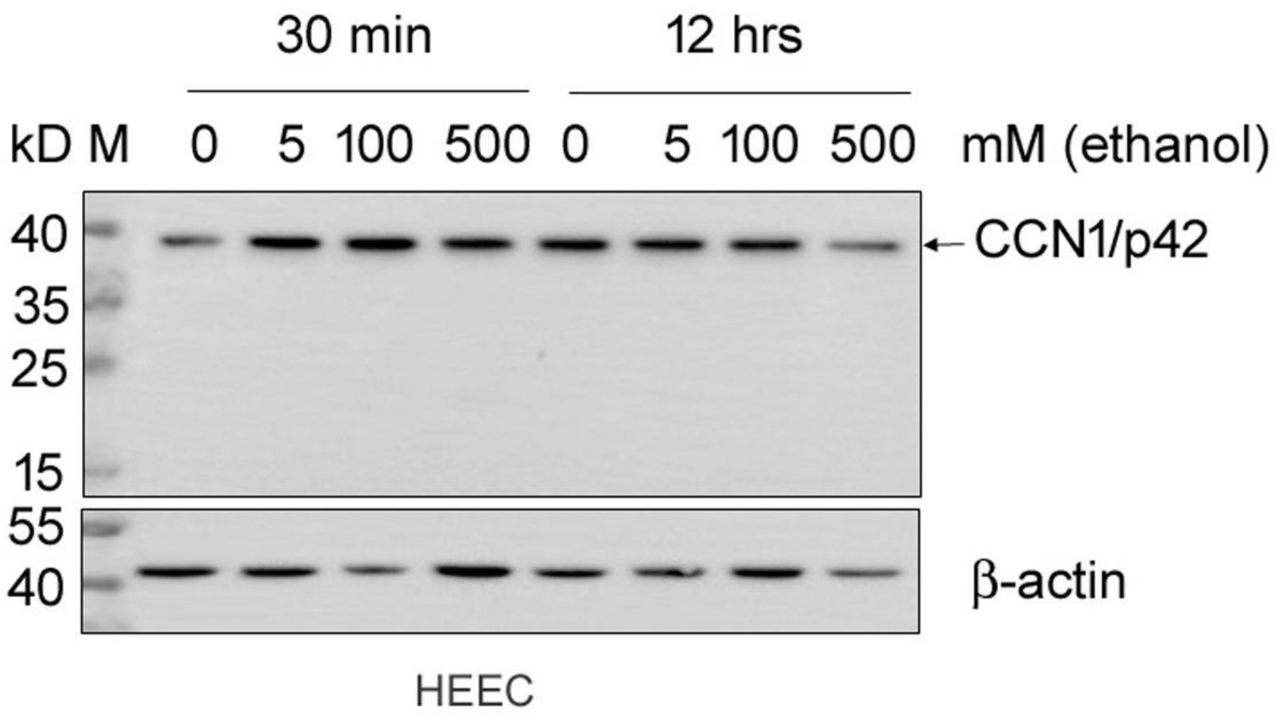

Fig. 2A. Western blot analyses of CCN1 expression in HEEC cells normalized to β-actin. The antibody for CCN1 was from OriGene (#TA349858).

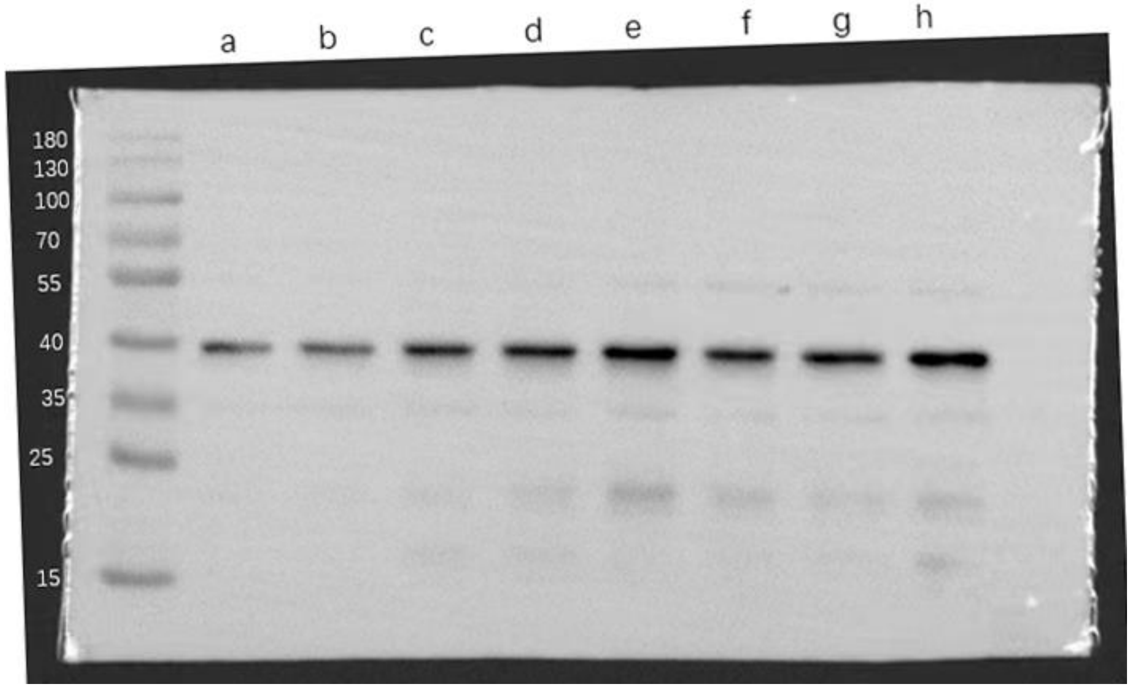

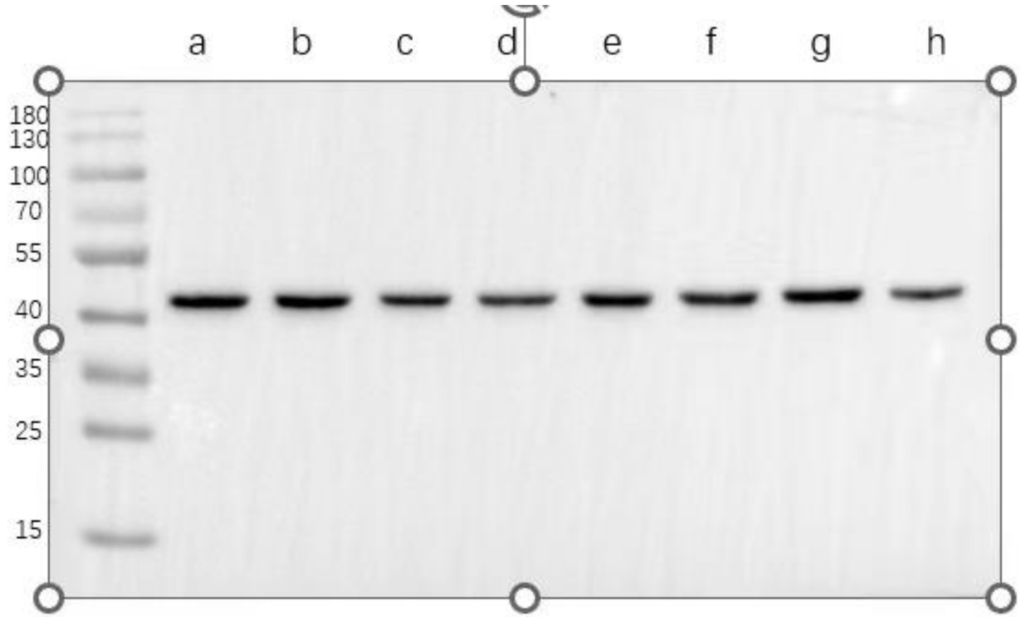

Originals in Fig. 2C. Western blot analyses of CCN1 expression in KYSE150 cells normalized to β-actin. The antibody for CCN1 was from OriGene (#TA349858).

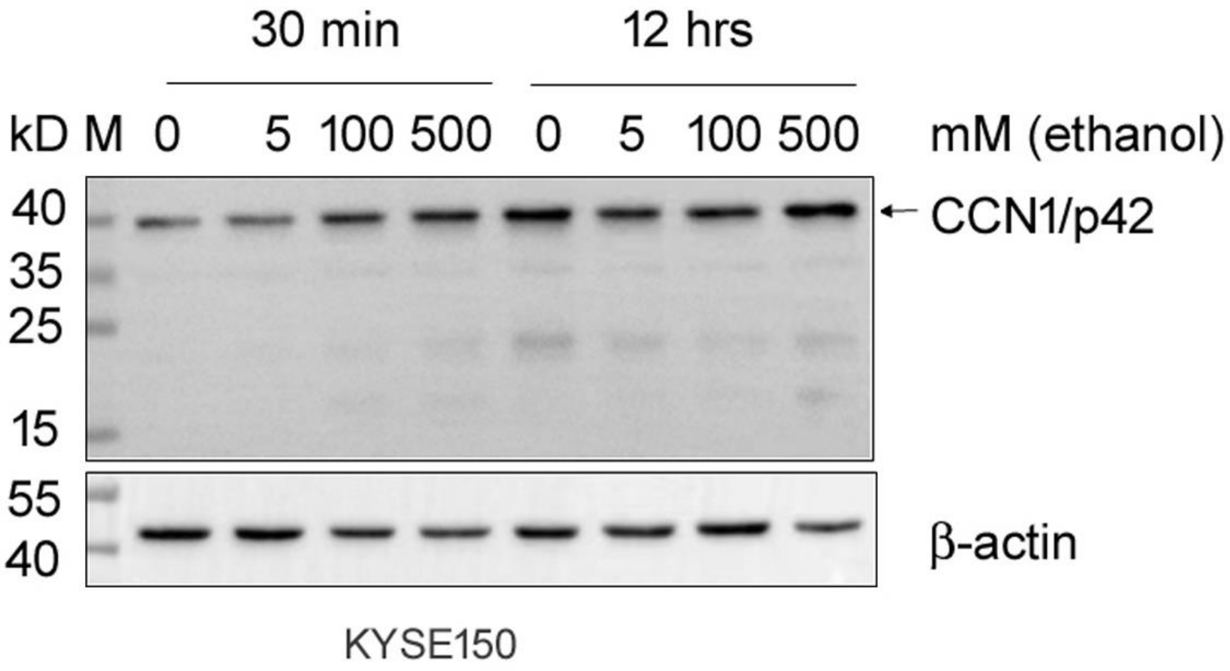

Fig. 2C. Western blot analyses of CCN1 expression in KYSE150 cells normalized to β-actin. The antibody for CCN1 was from OriGene (#TA349858).

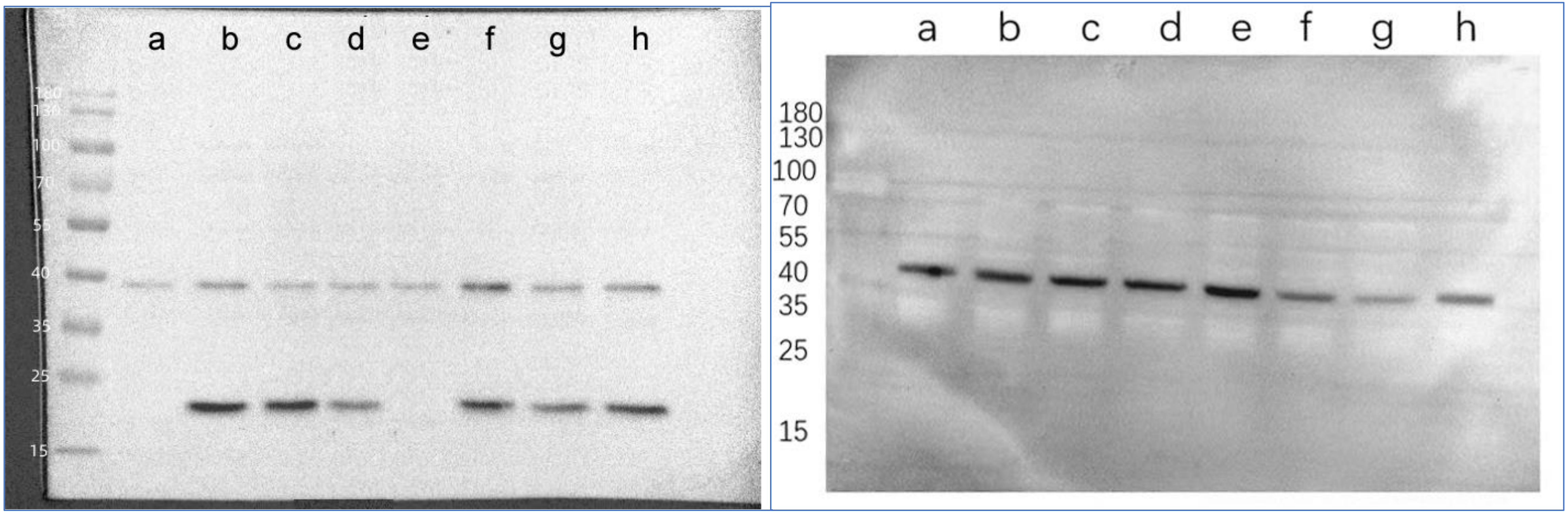

Originals in Fig. 2E. Western blot analyses of CCN1 expression in OE19 cells normalized to β-actin. The antibody for CCN1 was from OriGene (#TA349858).

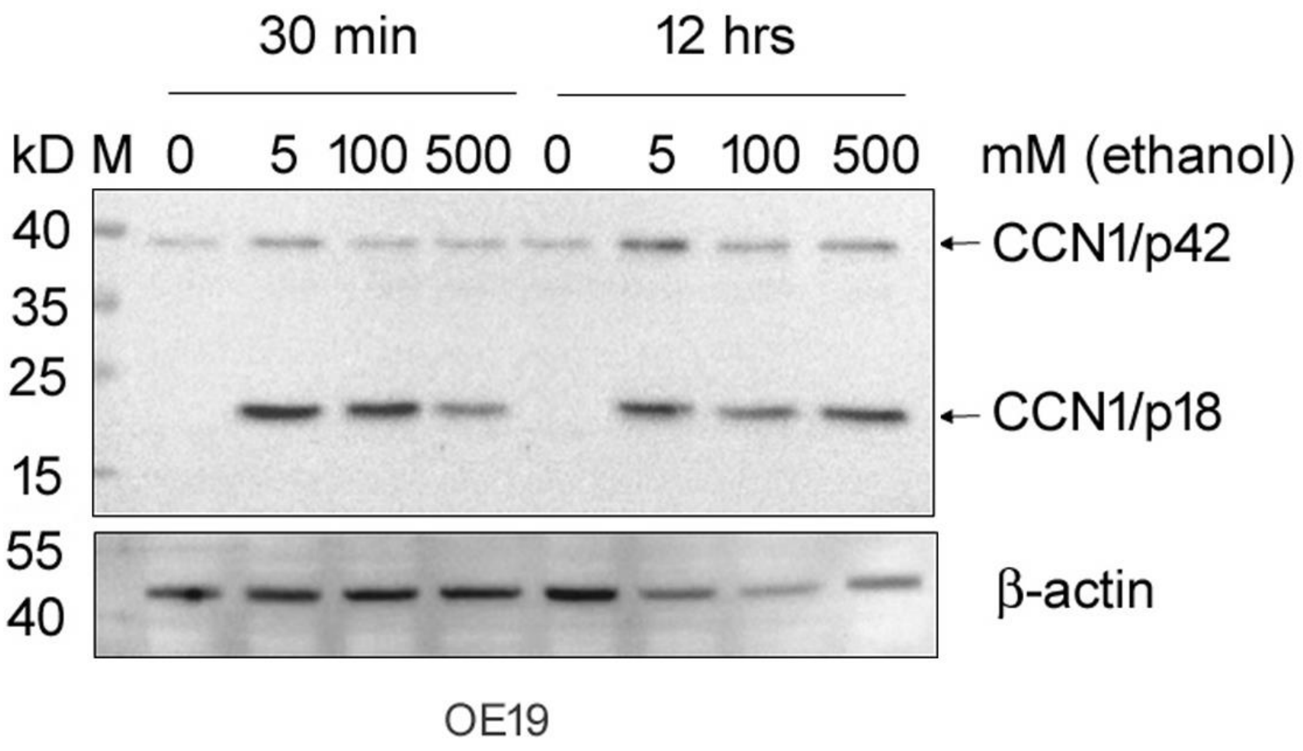

Fig. 2E. Western blot analyses of CCN1 expression in OE19 cells normalized to β-actin. The antibody for CCN1 was from OriGene (#TA349858).

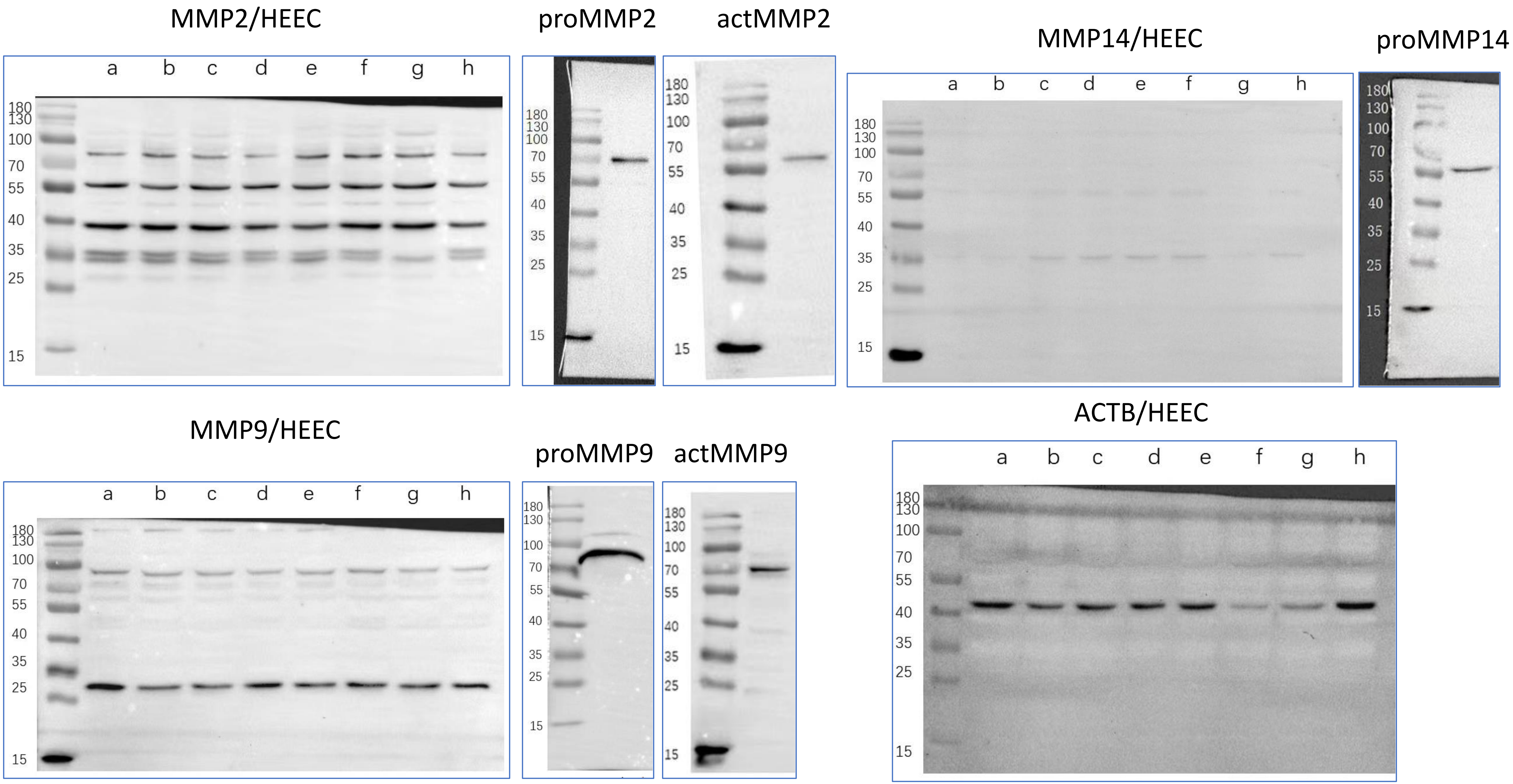

Originals for Fig. 3A. Western blot analyses of MMP2, MMP9, and MMP14 expression in HEEC cells normalized to β-actin. The recombinant proteins were posted on the side to indicate the location of the target protein.

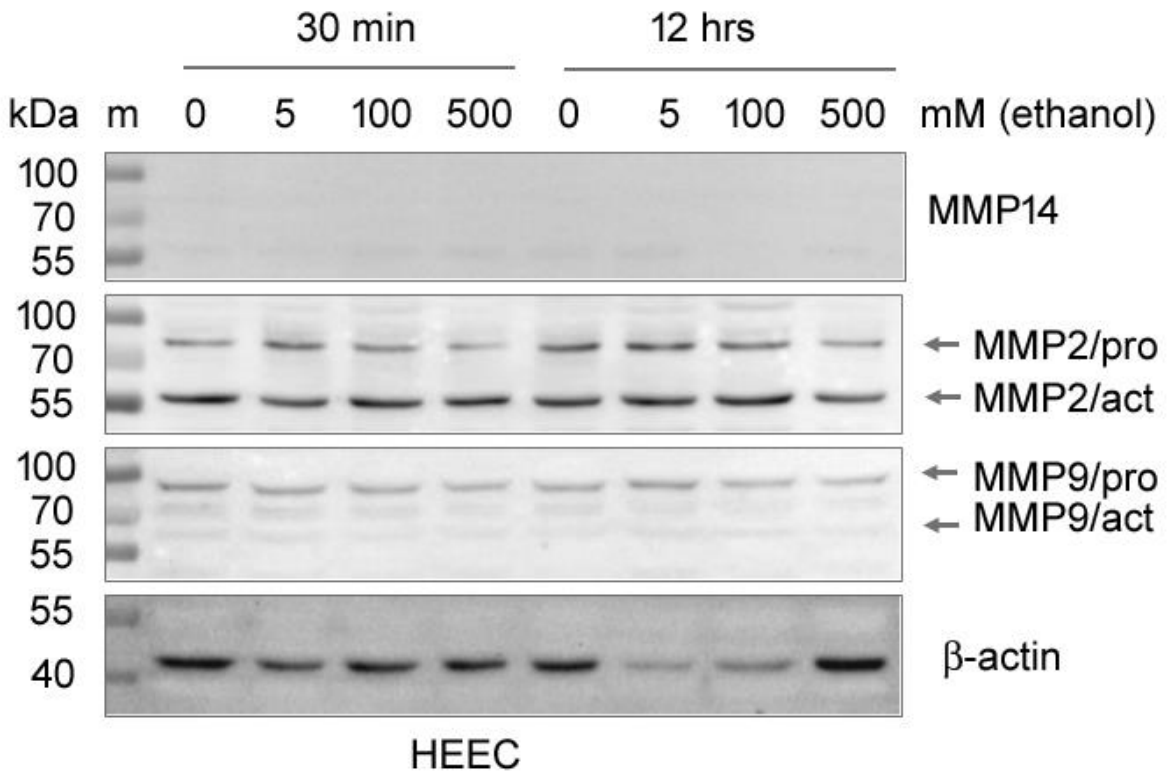

Fig. 3A. Western blot analyses of MMP2, MMP9, and MMP14 expression in HEEC cells normalized to β-actin.

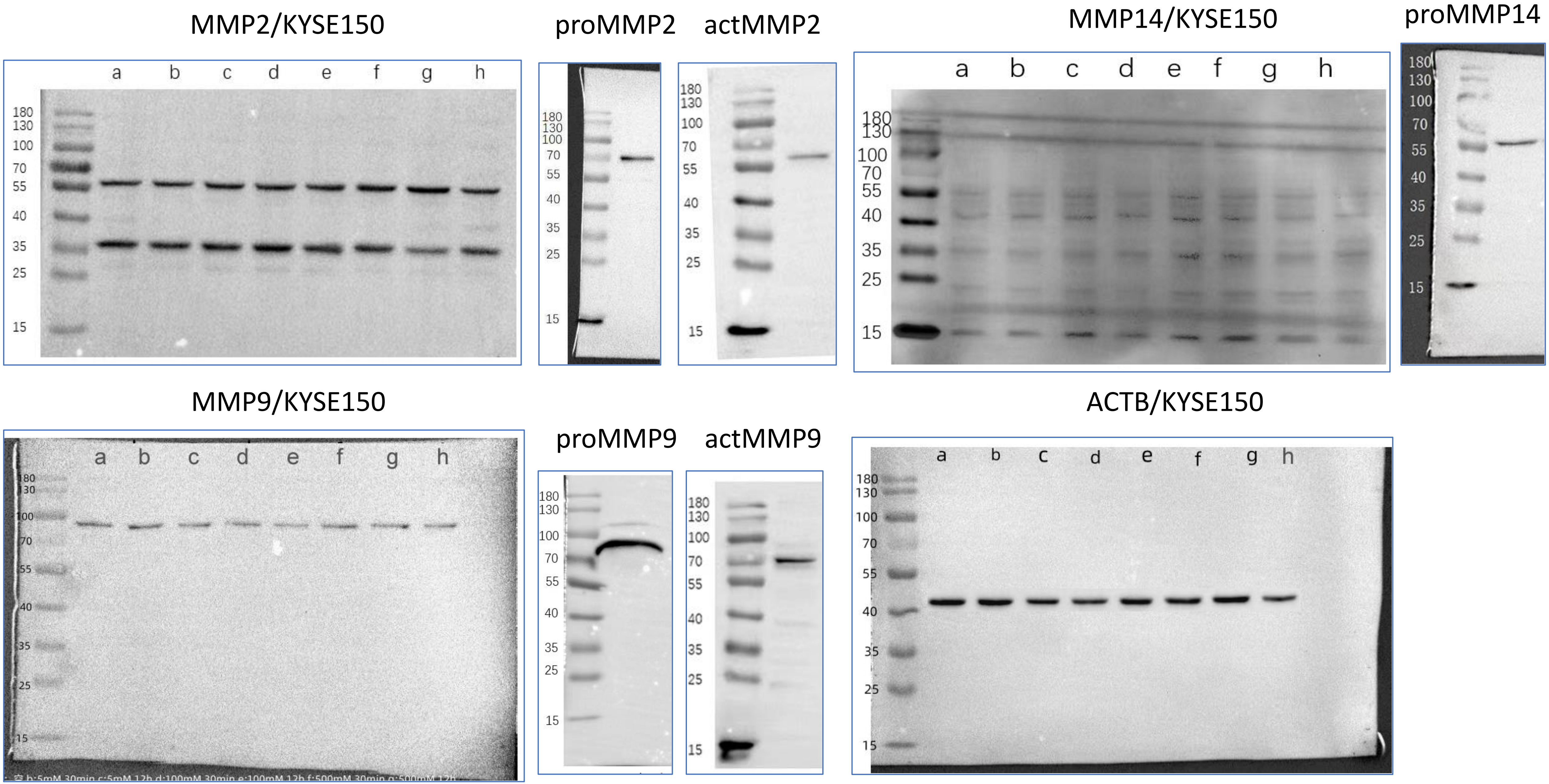

Originals for Fig. 3C. Western blot analyses of MMP2, MMP9, and MMP14 expression in KYSE150 cells normalized to β-actin. The recombinant proteins were posted on the side to indicate the location of the target protein.

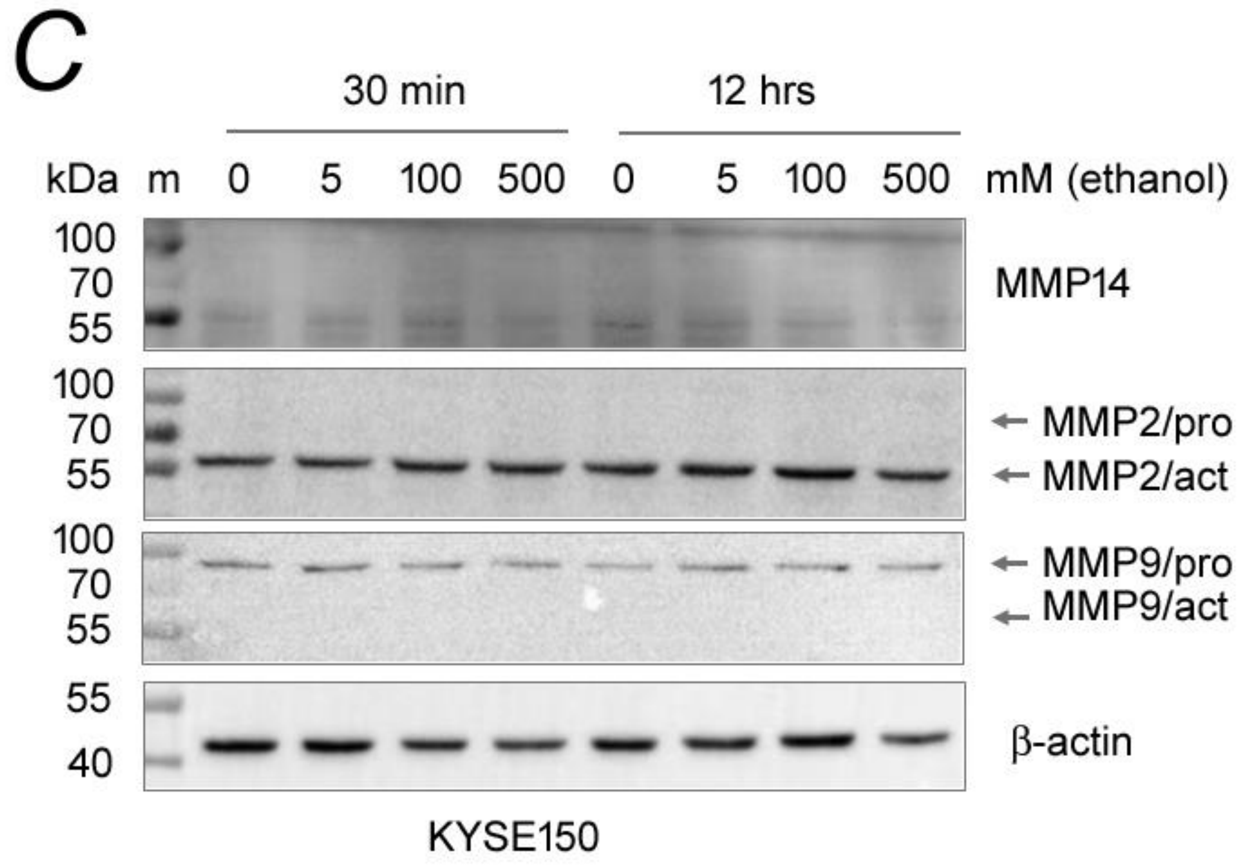

Fig. 3C. Western blot analyses of MMP2, MMP9, and MMP14 expression in KYSE150 cells normalized to β-actin.

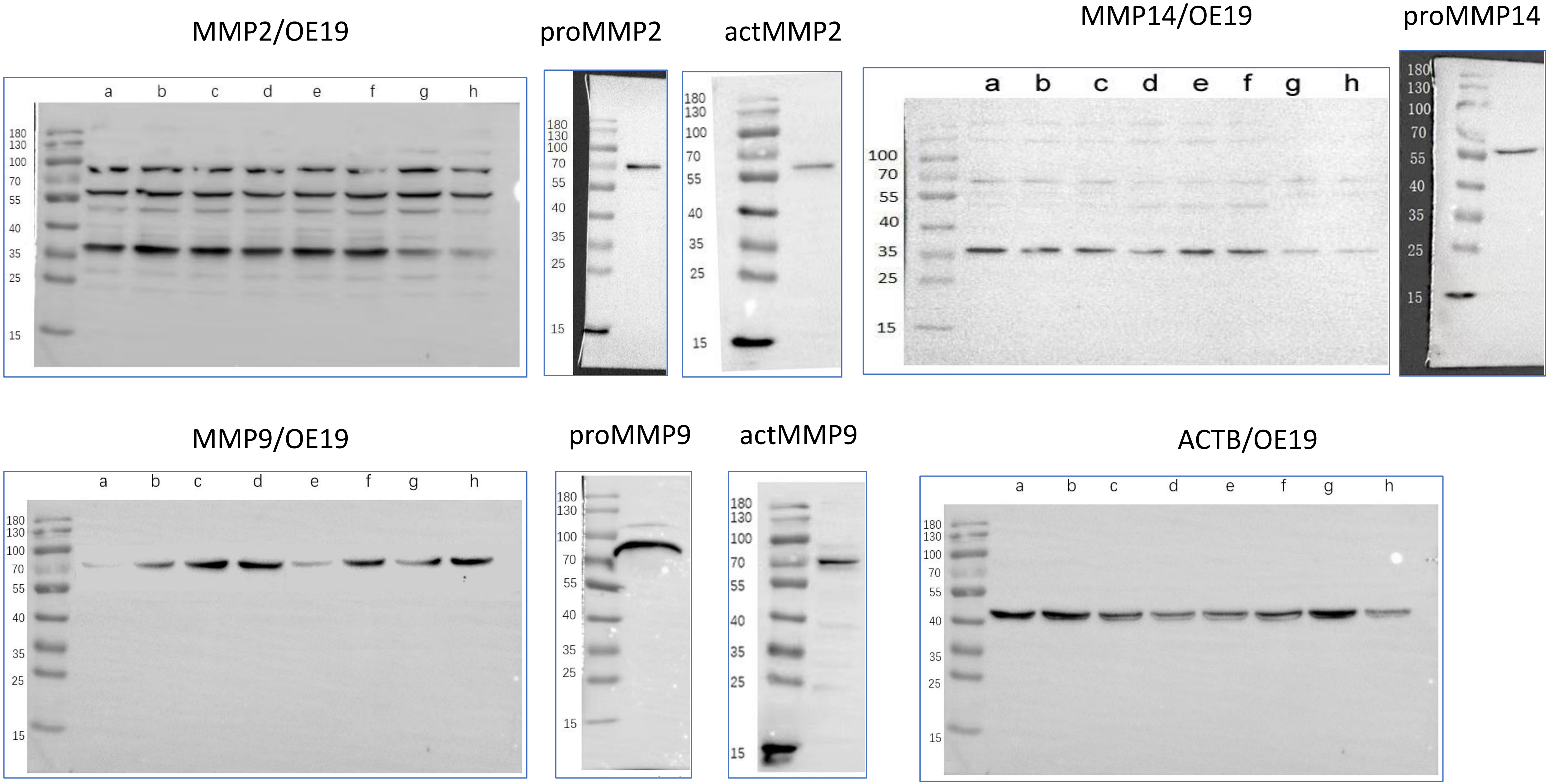

Originals for Fig. 3E. Western blot analyses of MMP2, MMP9, and MMP14 expression in OE19 cells normalized to β-actin. The recombinant proteins were posted on the side to indicate the location of the target protein.

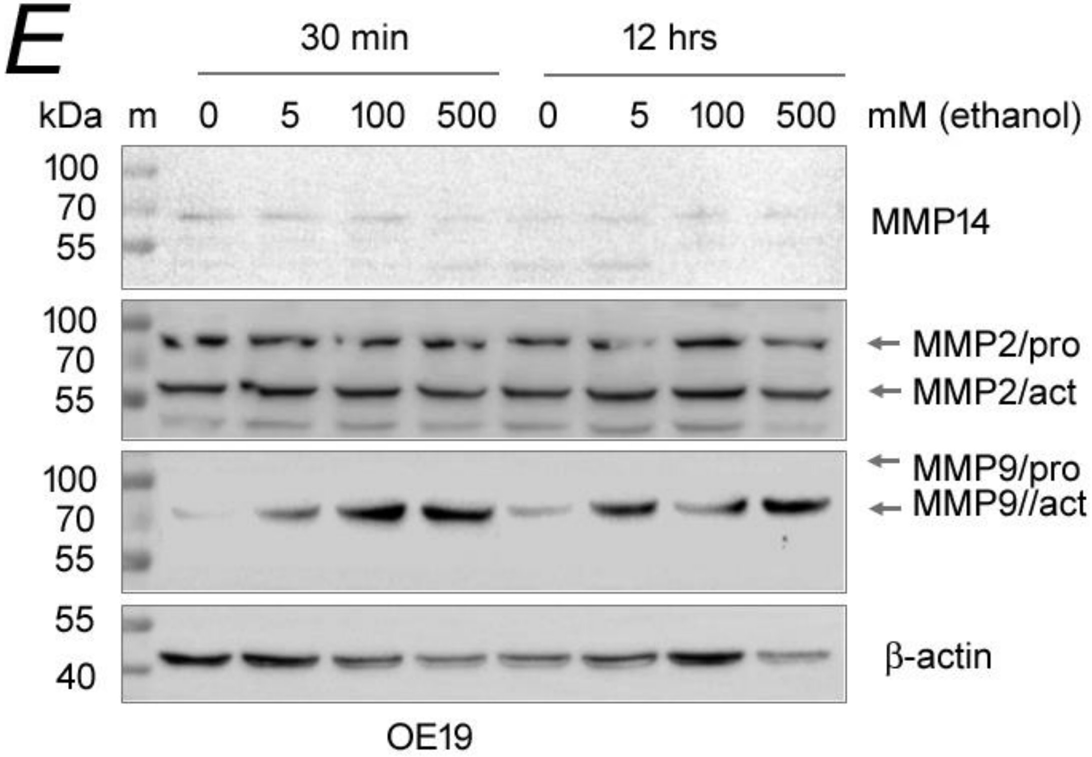

Fig. 3E. Western blot analyses of MMP2, MMP9, and MMP14 expression in OE19 cells normalized to β-actin.

CCN1 in Fig 5C. CCN1_LS in Fig 4C.

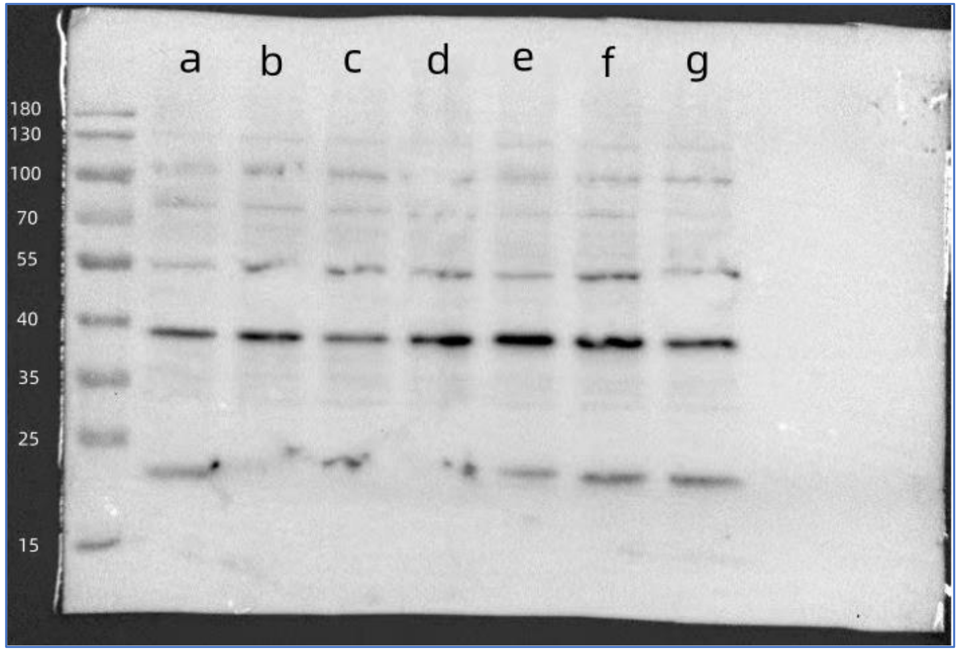

CCN1_SC in Fig 4C.

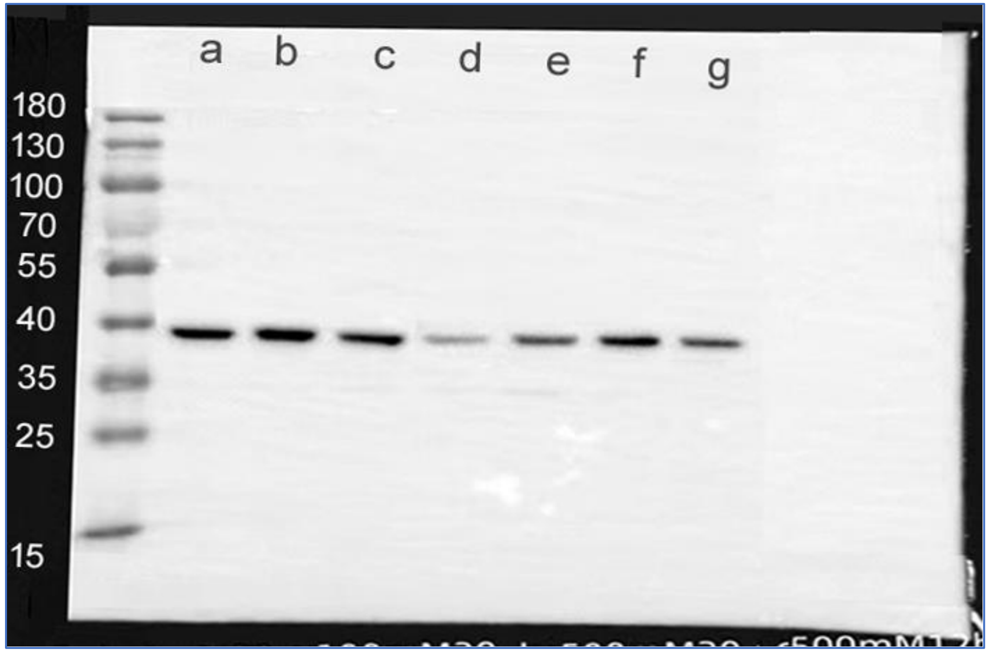

ACTB in Fig 4C.

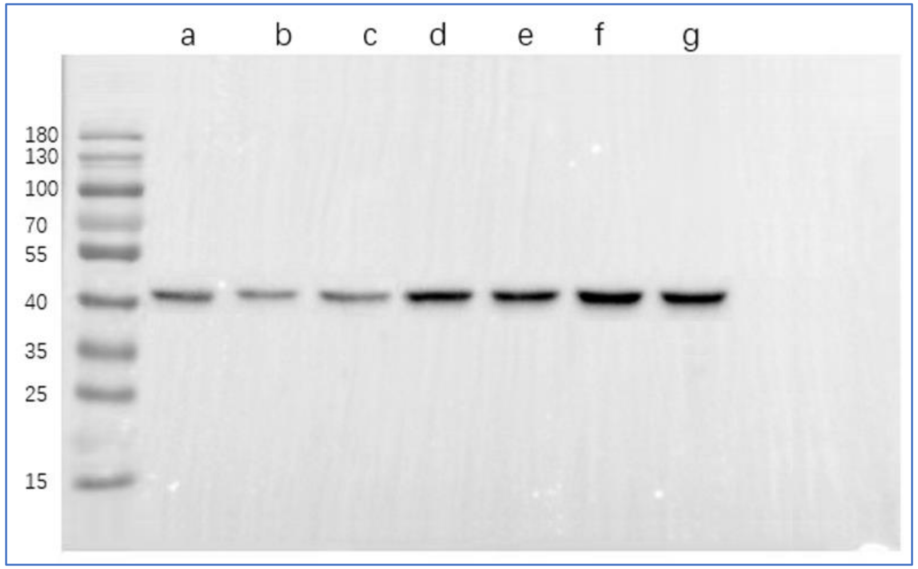

Originals in Fig. 4C/5C: After a 30-minute exposure of OE19 cells to various concentrations of ethanol, protein was extracted from the cells for Western blot analysis. Two CCN1 antibodies were used: the LS-C392953 antibody (upper left) recognizes the VWC region of CCN1, and the sc-271217 antibody (upper right) detects the CT region. ACTB was used as a loading control (lower left).

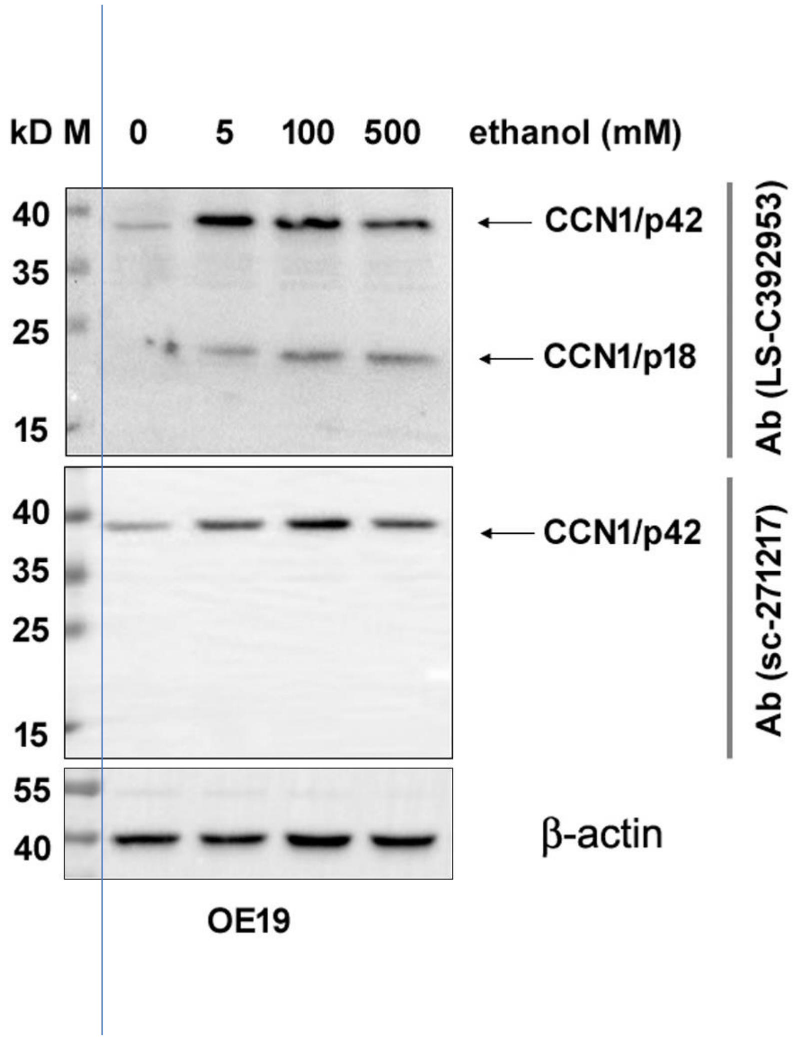

Fig 4C. After a 30-minute exposure of OE19 cells to various concentrations of ethanol, protein was extracted from the cells for Western blot analysis. Two CCN1 antibodies were used: the LS-C392953 antibody recognizes the VWC region of CCN1, and the sc-271217 antibody detects the CT region. The vertical line indicates the location of the merge.

CCN1 in Fig 4D. CCN1 in Fig 4E.

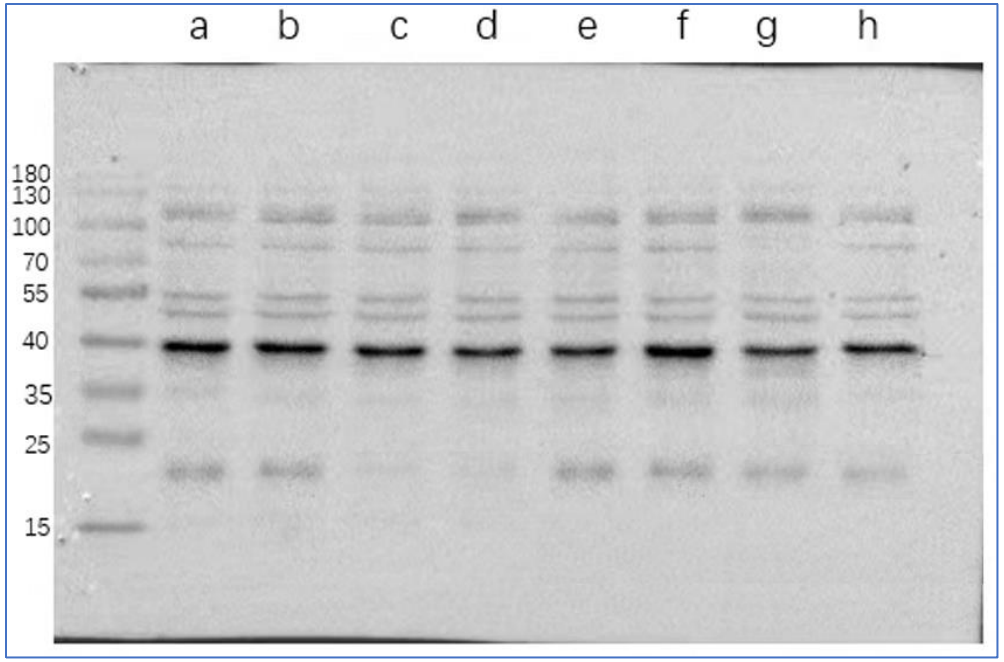

MMP9 in Fig 4D.

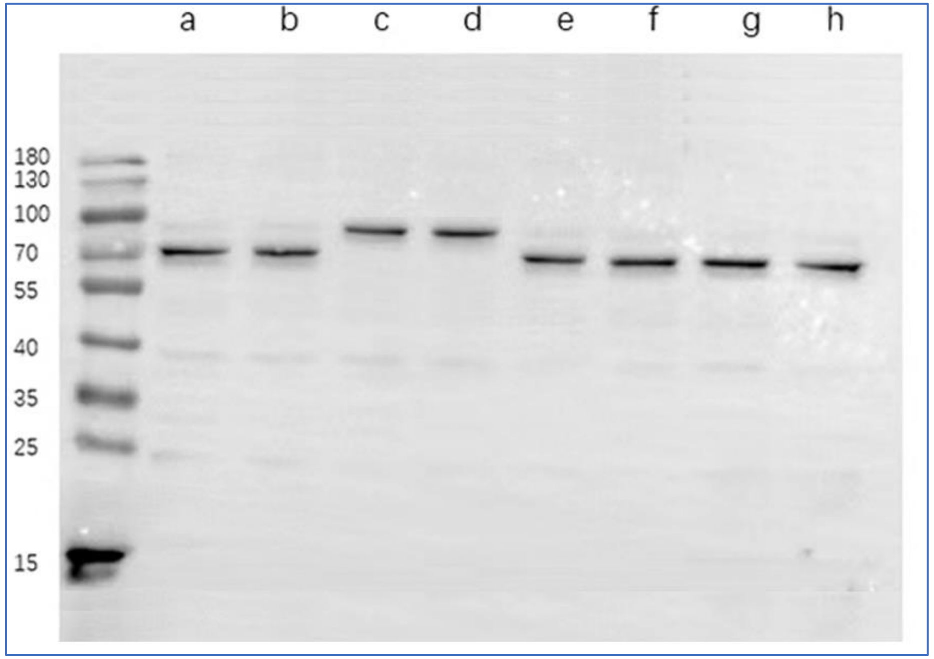

MMP2 in Fig 4E.

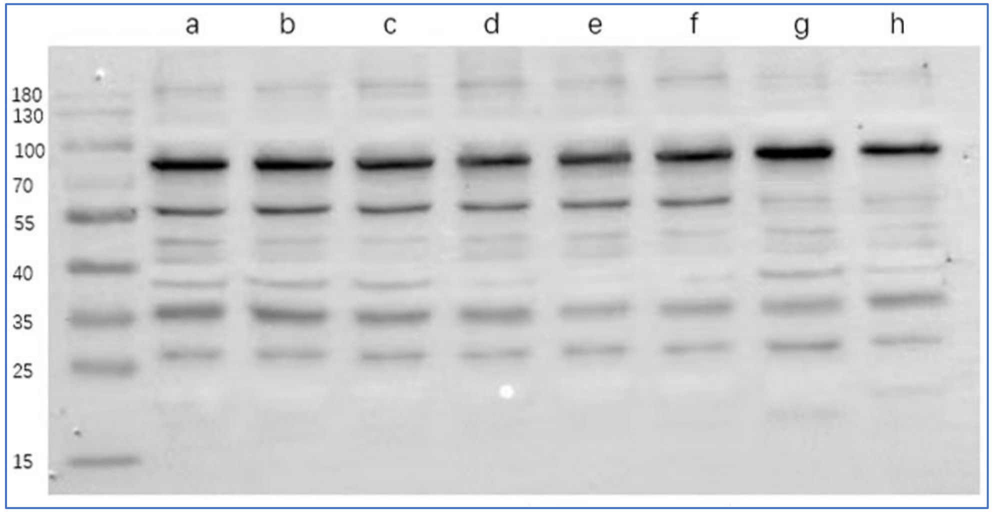

ACTB in Fig 4D. ACTB in Fig 4E.

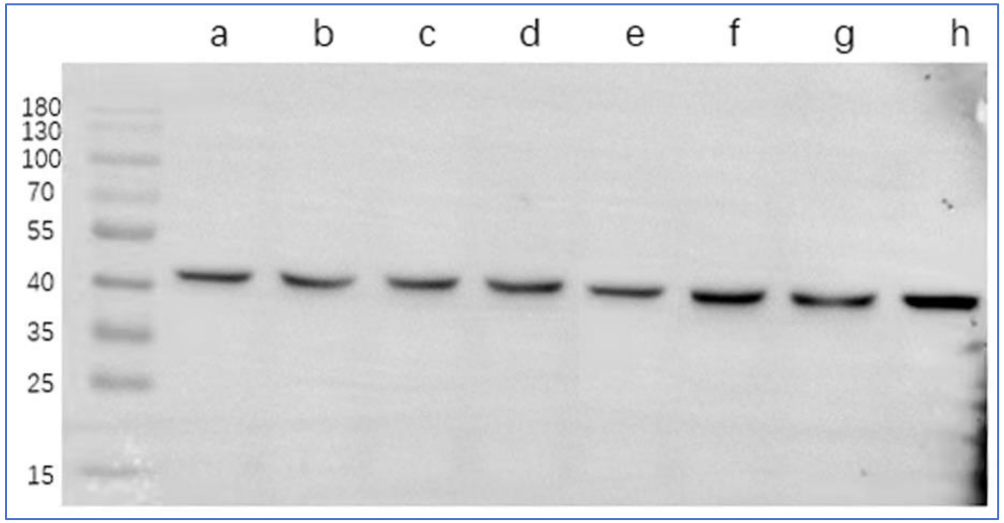

Originals in Fig. 4D/E/F: OE19 cells were pre-incubated with MMP9 inhibitor II before alcohol exposure. Protein was isolated from cells for Western blot analyses of CCN1 (#TA349858, upper left) and MMP9 (#TA378586, upper right), and normalized to β-actin (). OE19 cells were pre-incubated with MMP2 inhibitor I before alcohol exposure. Protein was isolated from cells for Western blot analysis of CCN1 and MMP2 (lower left), and normalized to β-actin (lower right).

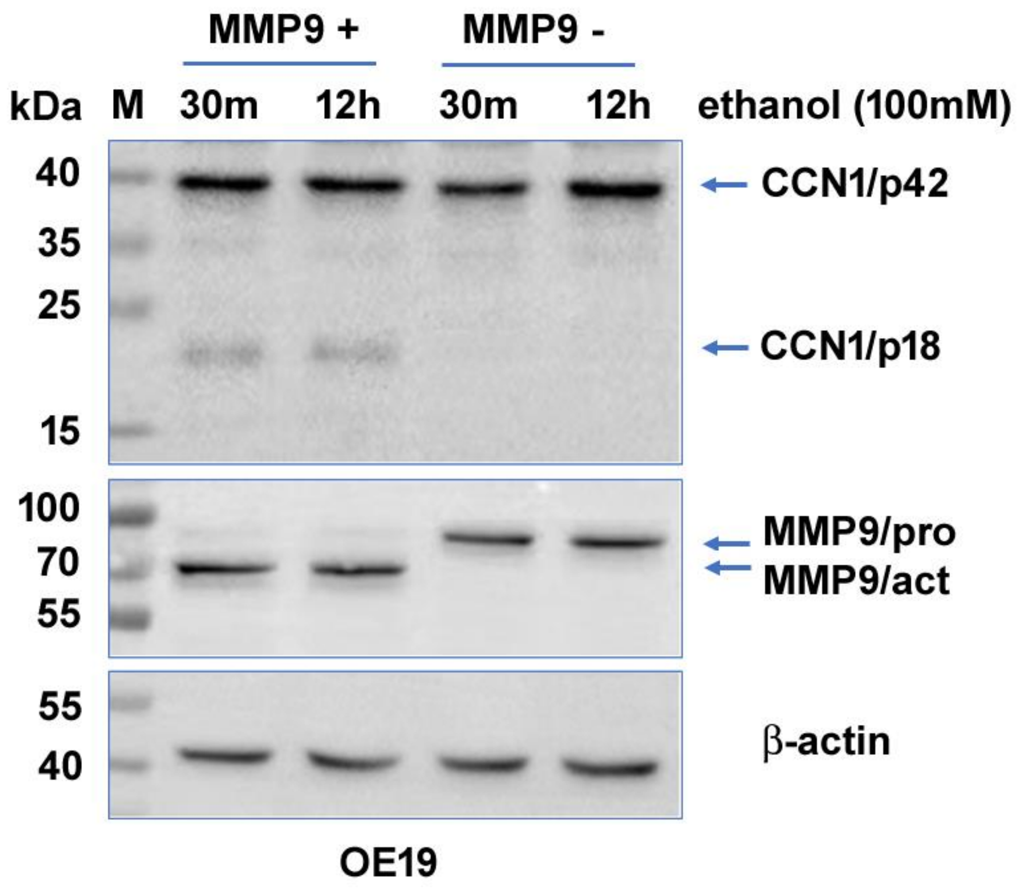

Fig 4D: OE19 cells were pre-incubated with MMP9 inhibitor II before alcohol exposure. Protein was isolated from cells for Western blot analyses of CCN1 (#TA349858) and MMP9 (#TA378586), and normalized to β-actin.

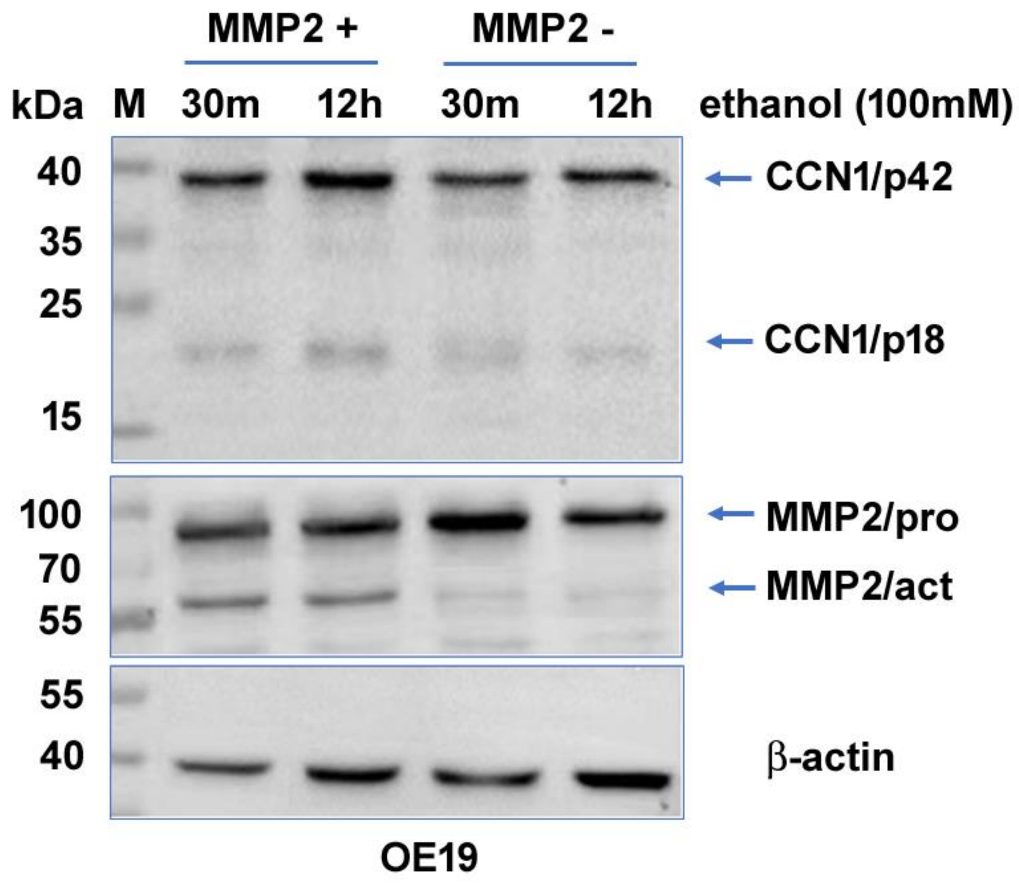

Fig 4E: OE19 cells were pre-incubated with MMP2 inhibitor I before alcohol exposure. Protein was isolated from cells for Western blot analyses of CCN1 and MMP2, and normalized to β-actin.

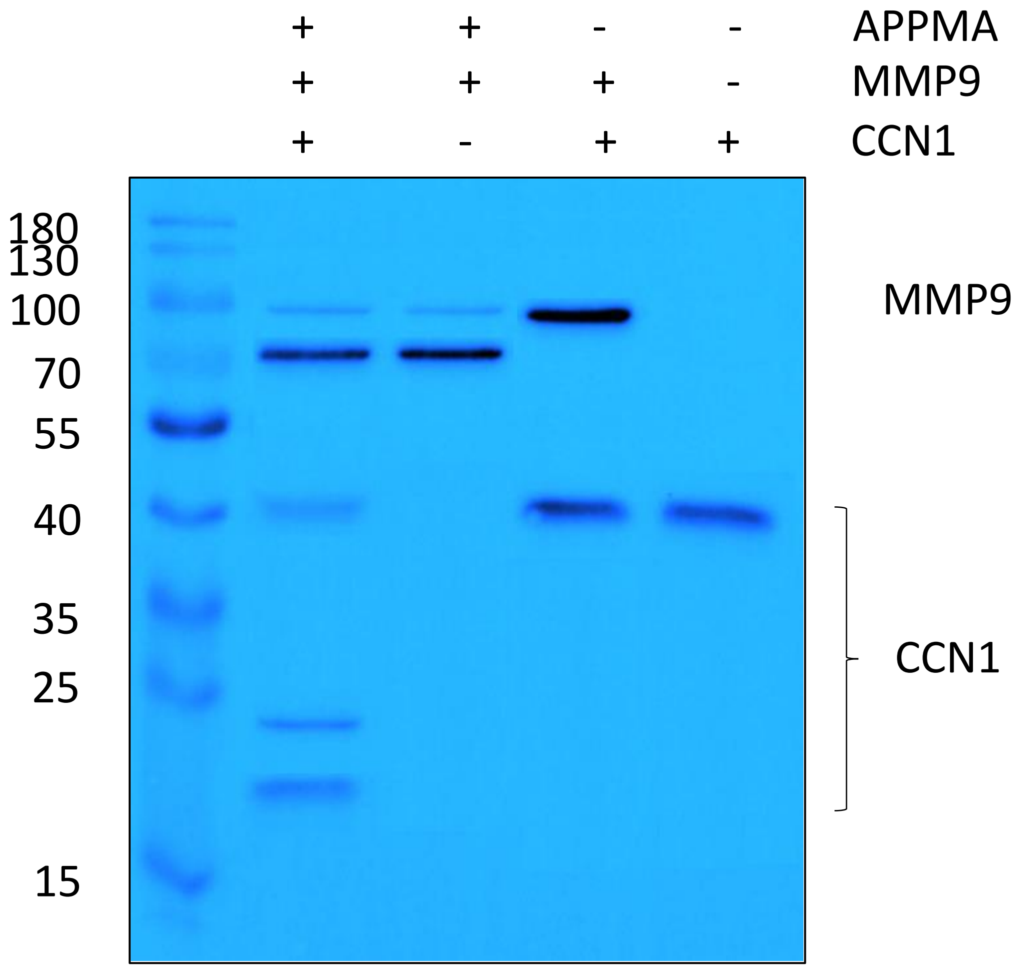

Original for Fig 5A: Cell-free assay of MMP9 and CCN1 interaction. A full-length recombinant CCN1 protein (rCCN1, #TP750169) was incubated at 1 μg/ml with a recombinant MMP9 (rMMP9, #911MP) at 0.4 μg/ml at 37 ˚C for 2 hours in the presence or absence of APMA. The gel was stained in 0.5% Coomassie Blue R-250 solution.

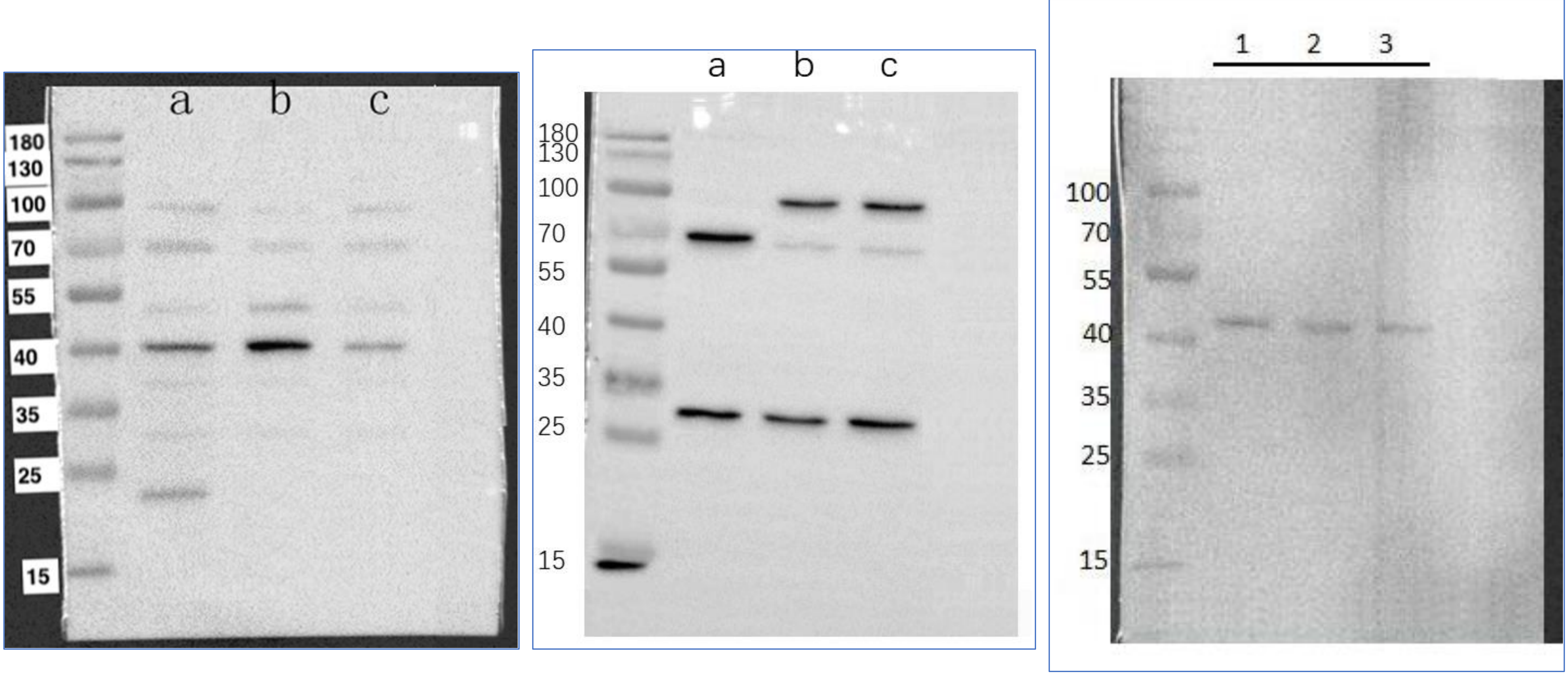

Originals in Fig 5B: HEEC cells were pre-incubated with APMA before alcohol exposure. Protein was isolated from cells for Western blot analyses of CCN1 (#TA349858, left) and MMP9 (#TA378586, middle), and normalized to β-actin (right).

CCN1 in Fig 5C.

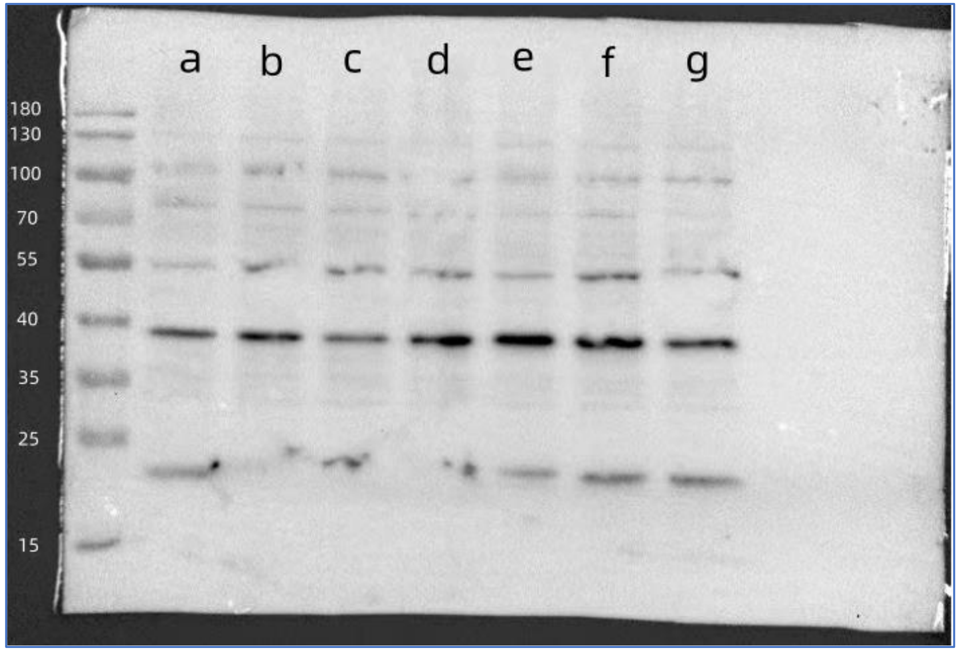

MMP9 in Fig 5C.

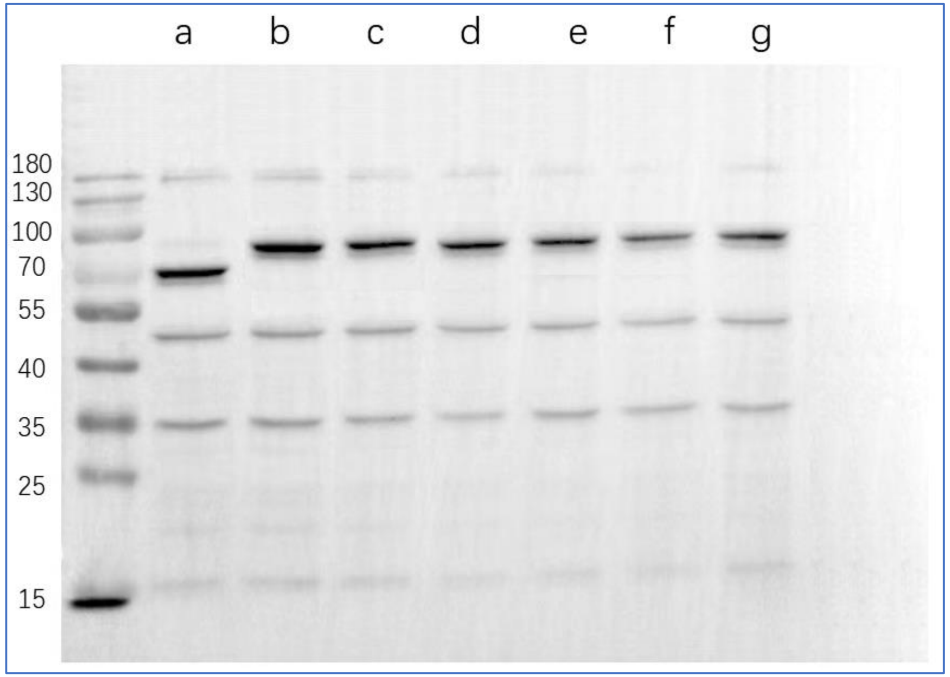

ACTB in Fig 5C.

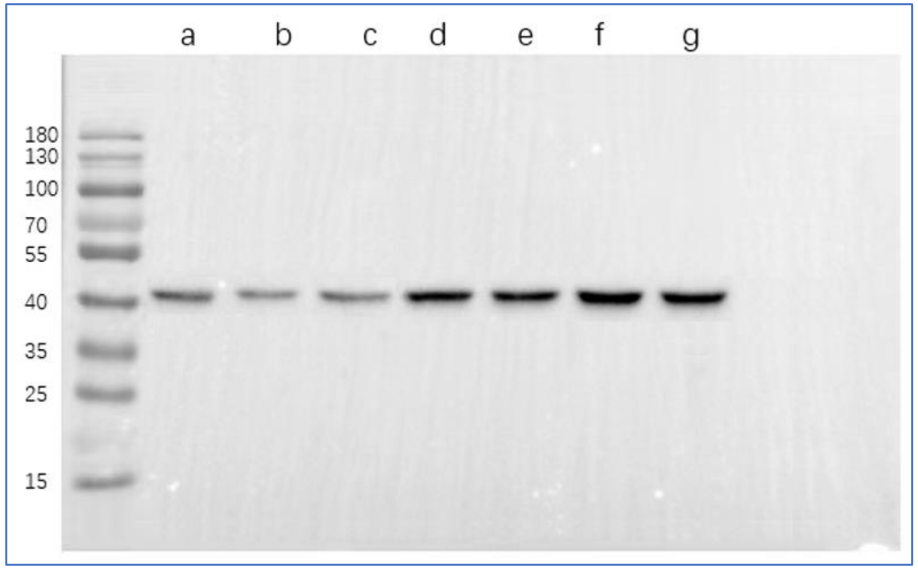

Originals in Fig. 5C: KYSE150 cells were pre-incubated with APMA before alcohol exposure. Protein was isolated from cells for Western blot analyses of CCN1 (#TA349858, upper left) and MMP9 (#TA378586, upper right), and normalized to β-actin (lower left).

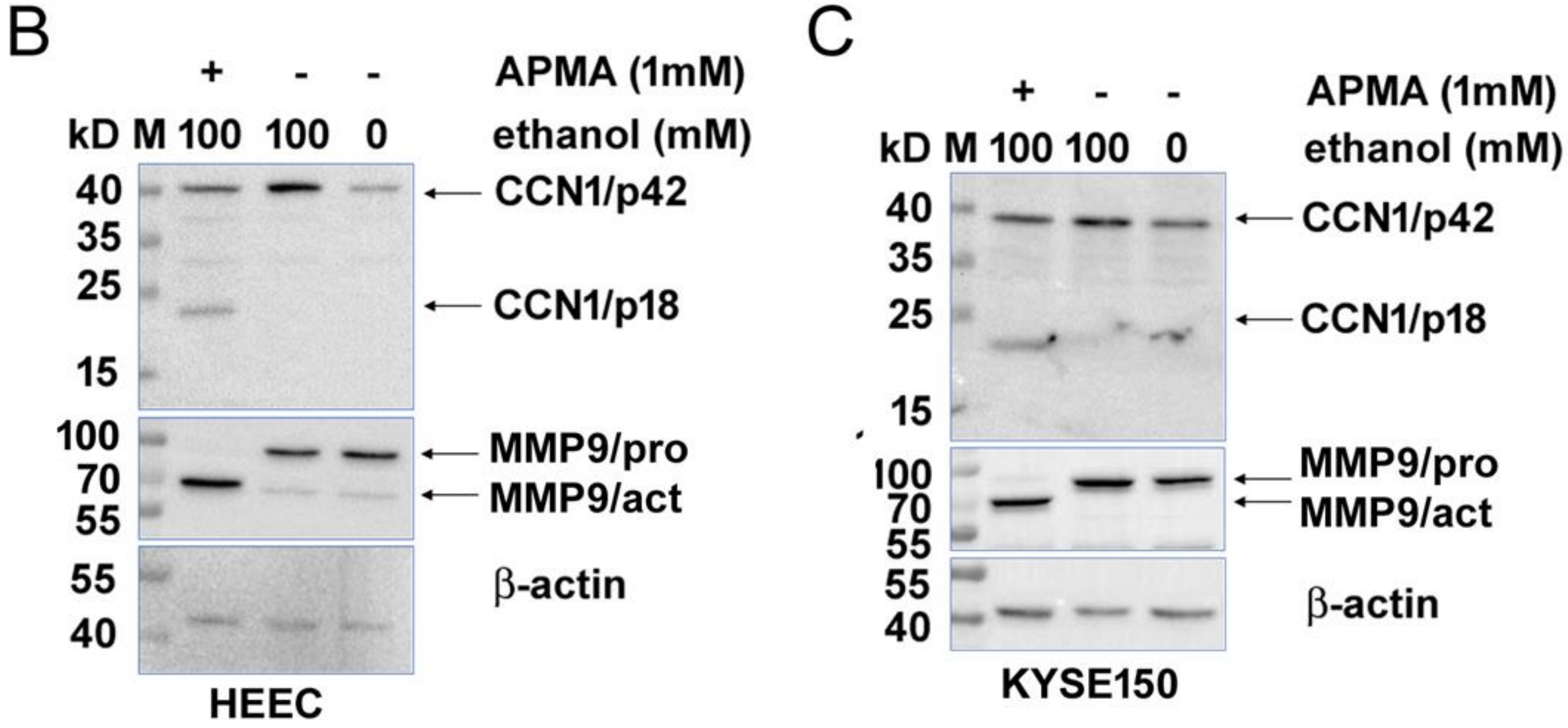

Fig 5B and 5C. HEEC cells were pre-incubated with APMA before alcohol exposure. Protein was isolated from cells for Western blot analyses of CCN1 (#TA349858) and MMP9 (#TA378586), and normalized to β-actin (left). KYSE150 cells were pre-incubated with APMA before alcohol exposure. Protein was isolated from the cells for Western blot analyses for CCN1 (#TA349858) and MMP9 (#TA378586), normalized to β-actin (right).
